# Genome Wide Adaptations of *Plasmodium falciparum* in Response to Lumefantrine Selective Drug Pressure

**DOI:** 10.1371/journal.pone.0031623

**Published:** 2012-02-27

**Authors:** Leah Mwai, Abdi Diriye, Victor Masseno, Steven Muriithi, Theresa Feltwell, Jennifer Musyoki, Jacob Lemieux, Avi Feller, Gunnar R. Mair, Kevin Marsh, Chris Newbold, Alexis Nzila, Céline K. Carret

**Affiliations:** 1 Kenya Medical Research Institute, Welcome Trust Research Programme, Kilifi, Kenya; 2 Nuffield Department of Medicine, John Radcliffe Hospital, University of Oxford, Oxford, United Kingdom; 3 Pathogen Microarrays group, The Welcome Trust Sanger Institute, Cambridge, United Kingdom; 4 Department of Statistics, University of Oxford, Oxford, United Kingdom; 5 Weatherall Institute of Molecular Medicine, John Radcliffe Hospital, University of Oxford, Oxford, United Kingdom; 6 Molecular Parasitology Unit, Instituto de Medicina Molecular, Lisboa, Portugal; Burnet Institute, Australia

## Abstract

The combination therapy of the Artemisinin-derivative Artemether (ART) with Lumefantrine (LM) (Coartem®) is an important malaria treatment regimen in many endemic countries. Resistance to Artemisinin has already been reported, and it is feared that LM resistance (LMR) could also evolve quickly. Therefore molecular markers which can be used to track Coartem® efficacy are urgently needed. Often, stable resistance arises from initial, unstable phenotypes that can be identified *in vitro*. Here we have used the *Plasmodium falciparum* multidrug resistant reference strain V1S to induce LMR *in vitro* by culturing the parasite under continuous drug pressure for 16 months. The initial IC_50_ (inhibitory concentration that kills 50% of the parasite population) was 24 nM. The resulting resistant strain V1S_LM_, obtained after culture for an estimated 166 cycles under LM pressure, grew steadily in 378 nM of LM, corresponding to 15 times the IC_50_ of the parental strain. However, after two weeks of culturing V1S_LM_ in drug-free medium, the IC_50_ returned to that of the initial, parental strain V1S. This transient drug tolerance was associated with major changes in gene expression profiles: using the PFSANGER Affymetrix custom array, we identified 184 differentially expressed genes in V1S_LM_. Among those are 18 known and putative transporters including the multidrug resistance gene 1 (*pfmdr1*), the multidrug resistance associated protein and the V-type H+ pumping pyrophosphatase 2 (*pfvp2*) as well as genes associated with fatty acid metabolism. In addition we detected a clear selective advantage provided by two genomic loci in parasites grown under LM drug pressure, suggesting that all, or some of those genes contribute to development of LM tolerance – they may prove useful as molecular markers to monitor *P. falciparum* LM susceptibility.

## Introduction

Chemotherapy is a key strategy in the control of malaria. To delay drug resistance, the current WHO recommendation is that different antimalarials be employed in combinations that include an Artemisinin derivative; this strategy is known as Artemisinin Combination Therapy (ACT). The first line treatment for malaria in many African countries is thus Coartem® which is composed of the Artemisinin-derivative Artemether (ART) and Lumefantrine (LM, Benflumetol) [1]. LM belongs to the quinoline-methanol (or aryl-amino alcohol) group of antimalarials structurally similar to Quinine (QN), Halofantrine (HLF) and Mefloquine (MFQ) [2]. Although Coartem® was hoped to reduce the speed of selecting drug-resistant parasites, there is growing concern that resistance may occur quickly, with LM perhaps even providing the main selective pressure due to its extended elimination half-life compared to that of the fast-acting ART [3].

Drug resistance is frequently linked to mutations and changes in the expression levels of transport proteins [4]–[9]. In *P. falciparum*, responses to quinolines and quinoline-methanols are associated with PfCRT (chloroquine resistant transporter) and PfMDR1. However, the exact mechanisms of drug action and development of resistance to this group of antimalarials remain poorly understood, and are sometimes contradictory. Although both chloroquine (CQ) and the quinoline-methanol MFQ target the parasite food vacuole, *in vitro* selection of MFQ resistance has been shown to result in an increase in *pfmdr1* copy number; at the same time an increase in CQ sensitivity was observed [10]–[12]. In South East Asian laboratory and field isolates, *pfmdr1* amplification to up to 5 copies is associated with decreased susceptibility to MFQ and LM [13]–[19]; on the other hand LM resistance in African isolates has been linked with CQ-sensitive PfMDR1 alleles (86N alone, or together with 184F, 1246D or 76K) [20]–[22], with no apparent *pfmdr1* copy number changes.

The selection of CQ-sensitive alleles during treatment with ART-LM suggests that this ACT could lead to the return of CQ susceptibility in endemic areas. Indeed, genetic analysis of Kenyan field isolates showed that parasites carrying wild type alleles at *pfcrt^76^* and *pfmdr1^86^* were the least susceptible to LM. However, we found these wild type isolates to display a wide range of responses to the drug (interquartile range IQR 93–202 nM) [23]. Therefore, *P. falciparum* responses to LM are probably not attributable to *pfcrt^76^*and *pfmdr1^86^* alone, but may involve additional genes, possibly transporters.

Although limited by the difficulty of obtaining stable, resistant phenotypes, *in vitro* selection of drug resistant *P. falciparum* is an important tool for identifying the genetic basis of antimalarial drug resistance [24]. The efficiency of *in vitro* selection can be enhanced by increasing the duration of *in vitro* culture but also through the use of high initial parasitaemias of multidrug resistant strains; such strains can acquire resistance at 10–1000 fold higher frequencies than sensitive strains, a phenomenon known as ARMD or accelerated resistance to multidrug [25], [26]. The majority of such *P. falciparum* selection studies with CQ, MFQ and Artemisinin, suggests that the selection of drug-resistant parasites occurs in 2 stages: the first phase gives rise to an unstable, drug-tolerant population followed by the selection of a truly stable phenotype. The initial lines arise relatively quickly within days to months and may be associated with gene amplification or deletion, or can result from the differential expression of various genes including the transporter PfMDR1 [26]. Changes in *P. falciparum* mRNA expression levels are often of low amplitude, but they are highly reproducible, dose-dependent and involve functionally related genes, indicating that they do represent physiologically relevant processes [27]. Therefore, at least some, perhaps many of the mechanisms associated with unstable tolerance arising from *in vitro* selection are similar to those that result in stable drug resistance in the field.

In yeasts and bacteria, whole genome approaches have proven valuable in the identification of key mechanisms underlying drug action and development of resistance [28]–[30]. Here we used *in vitro* drug selection to explore the mechanisms of LM resistance in the human malaria parasite *P. falciparum*. We cultured the *P. falciparum* isolate V1S – a multidrug resistant reference isolate highly sensitive to LM – for 16 months (166 asexual cycles) under varying, but steadily increasing concentrations of LM. At the end of the selection regimen we used global transcriptome profiling of the resulting drug-tolerant parasite isolate V1S_LM_ and the sensitive, parental V1S isolate to identify transcriptional changes that arose during this medium-term selection regime. Our results showed that the altered response of V1S_LM_ to LM during the establishment of this initial, unstable drug resistant phenotype was associated with clear changes in expression levels of distinct genes; these include known and putative parasite transporters and genes encoding for components of fatty acid metabolism. Clearly, additional genes (apart from PfMDR1 and PfCRT) may contribute to the acquisition of LM tolerance and resistance *in vitro*, and if validated, should be valuable molecular markers to monitor susceptibility to LM and possibly other antimalarial drugs in the field.

## Results

### *In vitro* Lumefantrine resistance (LMR) selection

**Figure 1:**
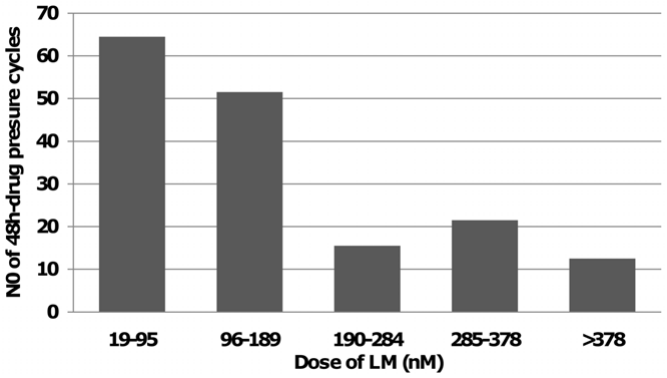
Lumefantrine drug selection regimen. Number of cycles during which V1S was cultured with varying Lumefantrine (LM) concentrations. In total, parasites were exposed to LM for 166 *P. falciparum* cycles, finally resulting in LM resistant V1S_LM_.

Drug resistant *P. falciparum* isolates can be generated *in vitro* by continuous culture in the presence of sublethal drug concentrations [26]. Particularly, multidrug resistant (MDR) strains show a high propensity to quickly adapt to drug pressure in culture [25]. Here, LMR was selected in the MDR reference strain V1S; V1S is fully resistant to CQ and antifolate antimalarials but highly sensitive to LM [31]. The V1S sensitive isolate displayed an initial half maximal inhibitory concentration, LM IC_50_ of 24±14 nM. After 16 months (166 *P. falciparum* cycles) of continuous culture under varying but increasing LM concentrations (Fig. 1) the selected parasite line V1S_LM_ grew in excess of 378 nM LM at a rate comparable to that of the parent isolate V1S; this is 15 times higher than the initial identified IC_50_. The LM IC_50_ of V1S_LM_ was elevated to 32±7 nM. The intraerythrocytic development cycle rates between the parent line (V1S) and the LM selected line (V1S_LM_) were similar using the statistical likelihood based approach described previously [32] (Fig. S1).When we finally cultured V1S_LM_ in drug-free medium, the IC_50_ returned to that of the initial, parental strain V1S within 2 weeks, indicating a clearly transient nature of resistance. After drug withdrawal, drug pressure had to be re-introduced again gradually in a dose escalating process starting from low concentrations and steadily increasing drug concentration as parasites got accustomed again to drug pressure.

### Gene expression profiling in untreated and drug selected parasites

Such rapid establishment of drug tolerance may rely on changes in the transcriptional repertoire of the parasite rather than mutations in specific genes. We sought to identify such adaptations of the transient LM-tolerant phenotype V1S_LM_. We performed global transcriptome profiling of V1S_LM_ compared to V1S by microarray analysis using the PFSANGER Affymetrix pseudo-tiling array [32]–[35]. To this end RNA samples from 3 biological replicates of V1S and V1S_LM_ cultures (selected as outlined above) were collected at 4 time points (0, 12, 24 and 36 hours) following synchronization with sorbitol. These samples correspond to ring forms, early and mature trophozoites, and schizonts. Out of the initial 24 samples (12 for each line) probed by microarray hybridization 19 were selected for final analyses as 3 showed signs of RNA degradation (Fig. S2A) and 2 displayed a median signal intensity that was out of range (Fig. S2B–D).

### Identification of differentially expressed genes in V1S_LM_ by Linear Modeling

**Figure 2:**
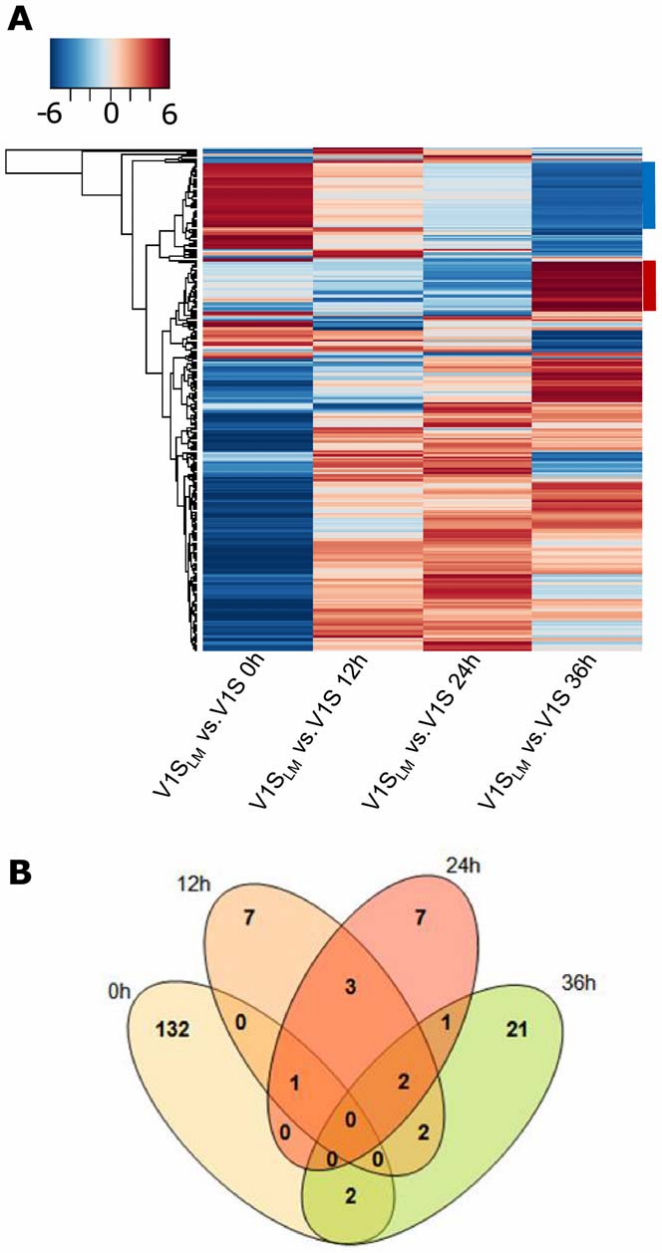
Changes in gene expression profiles between LM resistant V1S_LM_ and LM sensitive V1S *P. falciparum*. **A.** Heatmap of 589 expressed genes showing Differential Expression (DE) in at least one time point (F adjusted *p*<0.05). The 2 clusters highlighted with blue and red bars on the right hand side of the heatmap correspond to subtelomeric genes gradually switched off in the presence of LM, and transporters and cell cycle regulators gradually turned on in the presence of LM, respectively. Log2 ratio of V1S_LM_ vs. V1S expression is indicated by the color key ranging from −6 (blue, under-expression) to 6 (red, over-expression) **B.** Venn diagram showing the asexual life cycle distribution of DE genes. Analysis was based on linear modeling using Limma package of R/Bioconductor.

A linear modeling approach [36] was used to compare gene expression profiles of V1S (control) and V1S_LM_ (drug selected) pair wise at each time point (0, 12, 24, and 36 hours) (Fig. 2); in order to analyze expression profiles across the erythrocytic cycle (and identify genes found to be DE across the entire time course) a supplementary analysis using the EDGE program [37] was employed.

After fitting a linear model to the microarray data, we performed a pair wise comparison of gene expression in V1S_LM_ compared to V1S at each individual time point. An overall F test of variance identified 589 genes out of the total of 5778 genes represented on the array with an adjusted P<0.05 (Fig. 2A); this corresponds to 10% of the total number of predicted *P. falciparum* genes. 63% (371/589) displayed a minimum log 2 expression level of 4 when averaged across all time points and both culturing conditions. 266 genes were DE with a minimum fold change of 1.5 (Table S1), while 184 were DE between V1S_LM_ and V1S in at least one time point (Bayesian B>0); a total of 5189 genes remained unchanged. Our analysis revealed a subset of transcripts with opposite expression patterns, i.e. transcripts that were gradually switched off across time (all subtelomeric and included 1 *stevor*, 19 *rifins* and 19 *var* genes) and transcripts that were gradually turned on across time (consisting of ATP and GTP-binding proteins, cell cycle regulators, kinases and phosphatase and 4 transporters; marked by lateral blue and red bars in Fig. 2A respectively). While the first cluster may simply be associated with *Plasmodium* asexual development rather than be directly attributable to the drug pressure [38], the latter cluster highlights the up-regulation of transporter proteins under drug pressure.

**Figure 3:**
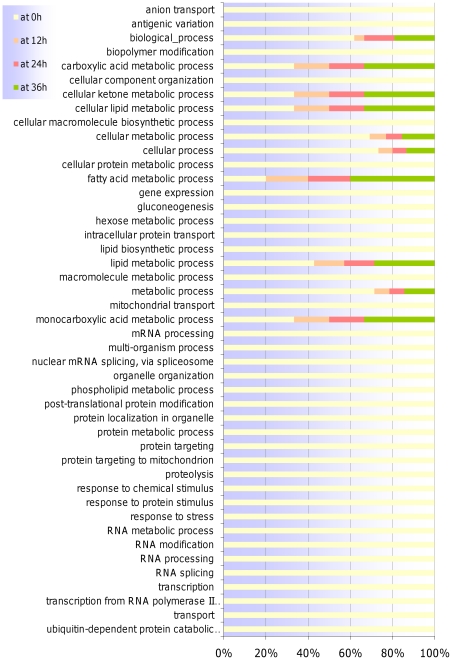
Gene Ontology (GO) analysis at main time points of the *P. falciparum* asexual life cycle. The time points color legend is indicated at the top left corner of the barplot. GO enrichment is indicated as stacked percentages.

Moreover, approximately ¾ (135/192) of all DE genes were seen at ring stage (0 h), while only 15, 14 and 28 emerged DE at 12 h, 24 h, and 36 h, respectively (Fig. 2B). The majority of DE genes were down-regulated at ring stage (101/144) while up-regulation was mainly observed in later time points (14/24 at 12 h, 12/14 at 24 h and 38/56 at 36 h). Among the most highly DE genes were those involved in antigenic variation (34, mainly at ring stages), transcription/translation (23), fatty acid biosynthesis (7) protein binding/folding (12), proteolysis (7), and those with a function in transport (14; annotated as such or containing at least 7 trans-membrane domains). Gene Ontology (GO) analysis (*P. falciparum* annotations downloaded in March 2010 at www.geneontology.org) (Fig. 3) revealed that at ring stage there was significant (P<0.05) over-representation of genes involved in transport activity [both by molecular function (MF) and Biological Process (BP)], phosphatidyl-inositol metabolic processes (BP), ubiquination (BP), and transferase activity (MF). At 12 h, genes involved in DNA polymerase activity were significantly over-represented whilst from 12–36 hours most of DE genes were those involved in fatty acid metabolic processes (BP).

### Known and candidate transporters are DE

Often, transporters are associated with drug resistance phenotypes [28]–[30]. 2.5% of all *P. falciparum* genes are predicted to encode transporter proteins with typically 7 or more (predicted) transmembrane (TM) domains; only few contain less than 7 [39]. Here we included those annotated as transporters (www.plasmodb.org) and proteins with at least 7 TM domains. Globally, our microarray analysis identified 18 candidate transporters (Table S2); linear modeling identified 14 genes, while the EDGE program highlighted a further 4.

Of those transporters identified by linear modeling, 8 were down-regulated at ring stage (including PfSR25/MAL7P1.64 which has been reported to be constitutively expressed in blood stages [40]), 1 up-regulated at late trophozoite stage, 4 and 1 were up and down-regulated in schizonts, respectively (Table S2, Table S1). After text mining and GO enrichment analyses, several of these identified transporters were of special interest: PfVP2 (PFL1700c), a folate/biopterin transporter (PF11_0172), the sugar transporter PFI0785c, the P-type ATPase PFC0840w and PfMRP1 (PFA0590w) were all over-expressed in late time points whilst PFI0720w (a putative 11 TM transporter of the Major Facilitator Superfamily) was clearly down-regulated at 36 h. PfVP2, sugar transporter PFI0785c, P-type ATPase PFC0840w, and PfMRP1 similarly displayed a gradual increase in expression during the intra-erythrocytic cycle in V1S_LM_ as compared to V1S (red marked cluster in Fig. 2A).

Four additional genes annotated to have transport functions were identified in our analyses; these included an ADP/ATP transporter on adenylate translocase (PF10_0366), the putative mitochondrial inner membrane translocases PF13_0300 and PF13_0358, and an oxoglutarate/malate translocator protein (PF08_0031).

The EDGE analysis identified 4 more genes containing 7 or more TM's: PFE1525w and PFL0220c (both conserved *Plasmodium* membrane proteins with unknown function), PFE0825w (a putative metabolite/drug transporter) and the putative sugar transporter PFE1455w.

**Figure 4:**
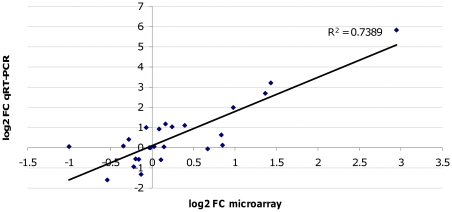
Correlation coefficients of log2 fold changes (FC) by RT-qPCR and microarray analysis. The linear regression is indicated on the plot, with an r^2^ = 0.7389.

From the 18 DE candidate transporter genes (Table S2), 5 were chosen for qRT-PCR analyses to confirm the microarray results (Fig. 4, Table S2). Transcript levels of PfMDR1 (PFE1150w), known to be associated with LM resistance, were also assayed and showed over-expression at 12 h. Although not significantly (F-test, *p* = 0.14), this transcript was over-expressed at 24 h in V1S_LM_ by microarray analysis. All significant genes identified by linear modeling were confirmed to be DE in at least one time point between V1S and V1S_LM_. Only 1 out of 10 genes (PFE1525w) identified by the EDGE analysis did not show differential expression by qRT-PCR.

### Genes involved in fatty acid metabolism

Interestingly, GO analysis revealed a significant over-representation of DE genes involved in fatty acid metabolism in V1S_LM_ (Fig. 3). Two of the transporters already identified (PFC0840w and PF08_0031) are predicted to be involved in phospholipid and dicarboxylic acid transport (BP and MF), respectively. Other genes identified are: PF13_0128 (a beta-hydroxyacyl-ACP dehydratase precursor), PF13_0285 (an inositol-polyphosphate 5-phosphatase), the phosphatidylinositol synthase MAL13P1.82, PF10_0016 (an acyl CoA binding protein isoform 2, ACBP2) and PF10_0015 (an acyl CoA binding protein, isoform 1, ACBP1).

### Chromosomes 2 and 10 ends are retained in long term *in vitro* culture

**Figure 5:**
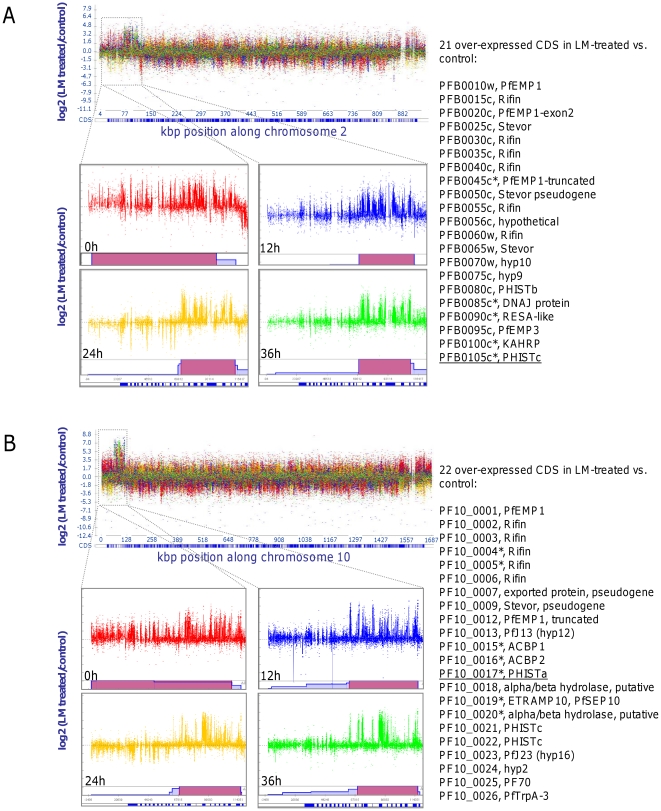
Chromosomes 2 (A) and 10 (B) show over-expression of contiguous probes covering 21 and 22 CDS, respectively. Amplification of the signals for the left arms of chromosomes 2 (**A**) and 10 (**B**) are enlarged for each time point as indicated. Every single coloured dot corresponds to a 25-mer probe: red is for 0 h, blue for 12 h, green for 24 h and yellow for 36 h. Underneath every enlarged chromosomal arm are pink bars indicating 100% robustness of signal amplification at *p*<0.01 (using SnoopCGH program with Smith–Waterman algorithm implementation). A normal distribution of the log ratios (y-axis) around the zero horizontal line is expected if the expression levels are the same along the chromosome (indicated as kilo base pair [kbp]). The CDS (represented under each chromosome by blue rectangles) contained within each amplified region are indicated on the right with their appropriate annotation (www.genedb.org). The genes marked with an * have been found significant at B>0 in the pairwise comparisons of the microarray data in at least one time point, while the underlined genes have been double checked by qRT-PCR.

Several consecutive genes on the left arms of chromosomes 2 and 10 showed consistent up-regulation in all time points in V1S_LM_ when compared to the parental isolate V1S; this observation was statistically significant for 6 and 7 of those genes on chromosomes 2 and 10, respectively (Fig. 5; Table S1). As *P. falciparum* gene expression is thought to be exclusively monocistronic [41], we speculated that the up-regulation is due to the loss of these loci in untreated parasites during culture. A segmentation algorithm, run at the probe level, confirmed clearly that the left arms of chromosomes 2 and 10 were amplified in V1S_LM_ compared to V1S (P<0.01), suggesting that the control line V1S could have lost these portions of the chromosomes after long term *in vitro* culture (Fig. 5A and B). For that reason we included PFB0105c (chromosome 2) and PF10_0017 (chromosome 10) to confirm the observed under-expression and possible deletion of the left arm of chromosomes 2 and 10 in V1S. Quantitative PCR (qPCR) using cDNAs and genomic DNAs isolated from V1S and V1S_LM_ at the end of the 16-month culture period showed a signal of amplification for PF10_0017 in the drug-resistant isolate while no signal was found in the parental line V1S. PFB0105c was 4–21 times more expressed in V1S_LM_ than in V1S, with a good degree of agreement between qRT-PCR and microarray analysis (global correlation coefficient r = 0.74, Fig. 4 and Table S3). Among the genes that were shown to be significantly DE by microarray and qPCR analysis are members of multigene families, including PHIST proteins (PFB0105c, PHISTc and PF10_0017, PHISTa).

## Discussion

In this work, we report the *in vitro* selection of lumefantrine (LM) resistance. We have selected a parasite line that grows in the presence of high LM concentrations, although its IC_50_ remains low. This feature has been observed with other antimalarials (mainly mefloquine) and we have discussed this phenomenon in detail [26]. This likely results from the methodology used to determining IC_50_'s (see Material and Methods) and the presence of more than one parasite population with different drug sensitivities. During drug selection resistant parasites will outgrow sensitive ones, while in the absence of drug, the opposite is observed (the resistant ones are outgrown by the sensitive ones); hence the observed low IC_50_. This highlights the limitation of current protocols of antimalarial chemosensitivity testing in detecting subtle changes in drug susceptibility *in vitro*, a limitation that has also been discussed elsewhere [42]. Nevertheless, such subtle changes most likely represent biologically relevant mechanisms as they may be similar to the changes occurring after exposure to sub-therapeutic levels of LM (during the long elimination phase of LM *in vivo*). It has been proposed that the exposure of parasites to such sub-therapeutic LM levels during this phase (in the absence of ART) provides the main selective pressure for resistance to Coartem® [3]. The unstable V1S_LM_ resistant phenotype we observed here is similar to previously reported phenotypes in antimalarial drug selection experiments [26]. More recently resistance to the potent antimalarial drug Artemisinin was described *in vitro* (associated with selection of dormant forms) [42] and *in vivo* (associated with prolonged parasite clearance times) but with no corresponding decrease in *in vitro* susceptibility to Artemisinin [42], [43]. However, as the growth rate of LM selected parasites was comparable to that of the parent line, the mechanism may not be similar to that observed for Artemisinin resistance *in vitro* in these studies [43], [44].

### Gene expression profiling and drug resistance

The utility of gene expression profiling to identify mechanisms of antimalarial drug action and resistance is controversial. Several studies reported low amplitude mRNA changes after *in vitro* exposure of parasites to CQ and antifolates with no apparent link to the drugs' presumed modes of action [44]–[46], while others observed biologically relevant responses after exposure to CQ [47], tetracyclines [48] or Artesunate [49]. However, despite low amplitudes these changes were dose-dependent and highly reproducible, supporting physiologically relevant processes involving functionally related genes [27]. Our results also show relatively small alterations in gene expression (2–4 fold at the most) after LM treatment, which could reflect tightly controlled mechanisms of gene regulation in malaria parasites in response to drugs. Alternatively, this may indicate that synergisms of multiple genes or compensatory mechanisms mediate the *P. falciparum* LM response rather than relying on a clear-cut, adaptive signature. In our study linear modeling analysis, non-hierarchical cluster analysis and GO enrichment of gene expression data highlighted genes associated with fatty acid metabolic processes and transport (7 and 14 genes respectively), and identified chromosomal changes between treated and untreated cultures. Of the 14 candidate transporters, 11 are annotated as transporters or translocators (www.plasmodb.org), and 8 had previously been associated with drug resistance, especially CQ [46], [47], [50]. Having been linked to drug responses (see below) support the notion that many of the newly identified genes here are indeed involved in LM tolerance. The repeated identification of similar genes using different methodologies strongly suggests that such adaptations are specific and biologically relevant; therefore they provide useful insights in understanding the response of *P. falciparum* to LM, and antimalarial drugs in general. As for the other genes, their localization and identification as part of the permeome, make them potential candidates for transporting drugs in and out of the cell [39].

### Transporter up-regulation after 16-month culture with LM

Parasites treated with sublethal CQ showed the putative P-type ATPase PfATPase7 (PFC0840w) and the V type-H+ pyrophosphatase PfVP2 (PFL1700c) to be DE [47]. Both genes were up-regulated in our analysis at 36 h. PfVP2 belongs to a novel class of plant and protozoan H+ pumps [51] and is localized to the digestive vacuole as well as the parasite plasma membrane [52], but was also found on the infected RBC membrane [53]. If the protein acts as an active pump responsible for the extrusion of protons from the parasite it would help maintain cytosolic pH balance together with PfATPase7, PfCRT and PfMDR1. This would explain the observed over-expression in response to LM here, and CQ in earlier studies.

We also observed an over-expression of *pfmdr1* (PFE1150w) in the LM-selected line V1S_LM_ by microarray analysis (albeit not statistically significant) and qPCR. *pfmdr1* copy number changes [13], [17]–[19] and point mutations [20], [21], [54], [55] are often associated with LM resistance, while increased *pfmdr1* transcript levels have been linked to quinoline-based drugs [49], [56], [57] and artemisinin [49], but not to the antifolate pyrimethamine. The up-regulation of *pfmdr1* may therefore be specific to only a certain group of antimalarials.

The ABC transporter *pfmrp1* (PFA0590w) was similarly up-regulated in mature parasites (24 h time point and later) consistent with its wild type transcriptional peak as measured by RNAseq [38]. The protein is localized to the plasma membrane and vesicles of trophozoite, ring and merozoite stages [58], [59], and is homologous to the human multidrug associated protein 2 (MRP2) [59], a major cause of cancer chemotherapy failure [5]. Deletion of *pfmrp1* in the CQ-resistant parasite W2 resulted in reduced parasite fitness *in vitro*, increased accumulation of radioactive glutathione and sensitivity to QN, CQ, piperaquine (PQ), MFQ and Artemisinin [58]. These effects may be attributed to an impaired ability to transport drugs and toxic metabolites suggesting that PfMRP1 is a molecular pump, which removes drugs and toxic metabolites from the parasite. Indeed, parasites in which PfMRP1 was disrupted accumulated higher levels of CQ and QN than wild type parasites [59]. The relatively small change in IC_50_ (2 fold) in PfMRP1 disrupted parasites in the cited study [58] suggests that PfMRP1 may rely on a functional interplay with other transporters in mediating drug resistance.

The expression levels of several transporters (PfMRP1, PfATPase7, PfVP2 and PFI0785c, Table S1) increased gradually as the erythrocytic cycle progressed from 0 to 36 h in V1S_LM_ (LM treated parasites) compared to V1S (control parasites), suggesting that the differential expression observed was indeed a consequence of LM pressure. Our microarray analysis also showed that 6 of the 18 transporters were up-regulated by at least 1.5 fold at mature stages (24 h–36 h, Table S2).This agrees with the proposed mode of action of LM which like other quinoline antimalarials is thought to interfere with heme polymerization which peaks in mature parasites [60], [61].

### Could increased fatty acid synthesis counteract impaired membrane recycling?

The quinoline-methanols MFQ and QN are known to bind with high affinity to membrane and purified phospholipids, suggesting that these molecules may indeed be biological targets [62]–[66]. These drugs are thought to exert their antimalarial effects by impairing membrane recycling within the endo-lysosomal system of the intracellular parasite [67]–[69], ultimately blocking heme degradation and causing death. LM is structurally similar to QN and MFQ. Therefore, increased fatty acids biosynthesis in V1S_LM_ parasites could compensate for impaired membrane recycling induced by LM pressure. An altered expression of few fatty acid metabolism genes (including PF10_0016 identified here) has also been reported after exposure of malaria parasites to CQ [70] and ART [49]. This observation further supports the view that analyzing the transcriptome can provide biologically meaningful information about the response of malaria parasites to drug.

### Retention of frequently lost chromosome ends provides a selective advantage during drug pressure

Deletion of approximately 100 kb of the left arm of chromosome 2 is known to occur spontaneously in laboratory and field isolates after short term culture [71]–[75]; our microarray data showed a clear over-expression of several consecutive genes from this locus in V1S_LM_ while V1S lost this segment of the chromosome. However, qPCR carried out on both cDNAs and genomic DNAs showed low levels of expression of PFB0105c in V1S, suggesting that the V1S line was not clonal and had already been deleted in some parasites. Long-term LM exposure subsequently resulted in the selection of parasites carrying this locus in the V1S_LM_ line. None of the genes from this locus have been linked to provide a selective advantage to grow under LM resistance. Similarly, 130 kb were deleted on the left arm of chromosome 10. Copy number variations (CNV) of 3 genes within this segment (PF10_0013, PF10_0014 and PF10_0023) have previously been reported in lab [73], [74] and field isolates [74] after short term culture. Four of the other over-expressed genes (PF10_0015, PF10_0016, PF10_0019 and PF10_0021) were found deleted in parasites treated with sub-lethal concentrations of CQ [70]. Our qPCR experiments detected no signal for PF10_0017 in the V1S parental line but clear amplification in V1S_LM_ using both cDNAs and gDNAs as templates, corroborating the absence in V1S but presence in V1S_LM_. The inverse relationship that has been described previously between CQ and amino alcohols such as LM [10]–[12], [23] may explain the opposite effects observed in this region of chromosome 10 whereby CQ pressure led to selection of parasites carrying the deleted genes in the cited study [70], whilst LM pressure led to selection of parasites carrying this region in our study.

Both segments of chromosomes 2 and 10 identified here carry the *Plasmodium* exported proteins (PHIST). Moreover, 5 other PHIST transcripts were found significantly DE in our analysis, prompting us to speculate that they may play a role in regulating the transport of substances and possibly drugs such as LM. However, further studies are needed to confirm the specific role of these gene deletions on chromosomes 2 and 10 in promoting LM resistance. Interestingly, a novel candidate gene (PF10_0355) was recently demonstrated to modulate resistance to LM and structurally related antimalarials, re-enforcing the notion that novel genes may indeed play an important role in antimalarial resistance (Van Tyne et al, 2011).

### Influence of genetic backgrounds on drug testing

We chose V1S (a multidrug resistant line sensitive to LM) as our parent isolate because *in vitro* multi-drug resistant isolates are known to acquire resistance faster than sensitive ones. Of course, the genetic background of the parasite can influence the acquisition of drug resistance [50], [76] and LM adaptations observed here may vary in other isolates. Here, the resultant V1S_LM_ resistant population grew steadily in high concentrations of LM, although the IC_50_ was only marginally increased and the phenotype ultimately transient. The comparison of these data with those obtained from additional tolerant or resistant isolates from diverse genetic backgrounds will shed further light on whether LM resistance varies according to the genetic background of the parasite. We compared the expression profile of V1S with that of the drug-selected line V1S_LM_ while under sub lethal concentrations of LM. A comparison of these expression data with those obtained from the V1S parent growing in the presence of LM at the time of sampling may provide insight into direct transcriptional effects of LM on parasites as opposed to the long-term effects of LM drug selection. Furthermore, beginning selection with a diverse mixture of parasites may increase the chances of selecting a parasite line with a genetic background against which stable resistance can develop.

In conclusion, this study has identified 18 candidate transport proteins (other than PfCRT and PfMDR1) that are potentially involved in modulating the LM response of *P. falciparum*. We also observed the over-representation of genes involved in fatty acid metabolism supporting the prevailing hypothesis that LM exerts its antimalarial action by interfering with membrane phospholipids. In addition, our data highlight an over-expression of consecutive genes on the left arms of chromosomes 2 and 10 (some of which were previously observed to be deleted in laboratory and cultured field isolates), suggesting that these genes may have a role, even if indirect, in mediating antimalarial drug resistance. Interestingly, an opposite effect of CQ and LM on several genes on the left arm of chromosome 10 was documented. This study demonstrates that the analysis of the expression profile of malaria parasites under drug pressure may provide biologically important insights on drug response that will be useful for the immediate field. More functional studies and the analysis of field isolates are required to clarify the exact function of the transport proteins, fatty acid metabolism genes and chromosomes 2 and 10 deletions in mediating resistance to LM and other antimalarials. Further validation is needed to confirm if the genes identified in our study can be used as robust molecular markers to track LM resistance in endemic settings.

## Methods

### Selection of drug resistance *in vitro*

To select LM resistant parasites *in vitro* we cultured V1S isolates (multi-drug resistant strain sensitive to LM) continuously under varying concentrations of LM. Initially, parasites were maintained in culture according to Trager and Jensen methods [77] at concentrations higher or equal to the IC_99_ (inhibitory concentration that kills 99% of parasites). Parasites were cultured in 30 ml medium in 75 cm^2^ culture flasks at initial parasitaemias of 5–10% and 4–6% haematocrit. This process was continued and the drug pressure varied after each 48 hr cycle depending on the parasitaemia and the viability of the parasites (observed under a microscope), i.e. when parasitaemia was <0.5% the drug pressure was reduced to as low as IC_10_, and then raised steadily to concentrations higher or equal to the IC_99_ once growth had resumed. To ascertain that growth had resumed, the parasites were maintained in culture under decreased drug pressure until parasitaemia had increased 2 to 3 fold. Thereafter ⅓ of the culture was subjected to another drug selection cycle. Parasites were scored as growing steadily in drug if growing exponentially in a drug concentration for 3 or more consecutive cycles. Parasites were cryopreserved at each selection cycle when growth rate had resumed. At first, the cycle between relieving the parasites of drug pressure and resumption of normal growth could last 2 to 3 weeks after which the growth rate increased steadily after a period of continuous drug pressure.

### IC_50_ determination

To assess the IC_50_ of the selected line, the culture medium was removed and the resulting pellet re-suspended in complete culture medium, thus relieving the parasites of drug pressure. The parasites were then maintained in routine culture over a period of 10–14 days in the absence of drug. Thereafter, the IC_50_ of the parasite was determined to assess the stability of the resistant phenotype.

### Chemosensitivity testing

LM was dissolved in 90% methanol plus 10% HCl. Chemosensitivity assays were carried out in 200 ul cultures containing 0.5% parasitaemia and 1.5% haematocrit in 96 well microtitre plates. The prepared test plates were incubated at 37°C in a humidified airtight box flushed with 3% CO_2_, 5% O_2_, 92% N_2_ mixture. After one cycle (48 hrs), 25 ul of tritiated hypoxanthine, (3H-hypoxanthine, 0.5 uCi) diluted in culture media was added to each well and incubated for a further 18 hrs. Well contents from each plate were then harvested onto glass fibre filter mats using a 96 well cell harvester (TomTec Inc and Perkin-Elmer) and the amount of ionizing radiation determined using a Wallac 1450 Microbeta counter. Results were expressed as the LM concentration required for 50% inhibition of ^3^H incorporation into parasite nucleic acid (IC_50_) obtained by non-linear regression of the dose response curve.

### Cultivation of parasites for microarray transcriptome analysis

Cultures of V1S (control) and V1S_LM_ growing steadily in 142 nM LM (a concentration 6× the IC_50_ of the parent line V1S) were maintained in 75 cm^2^ culture flasks at 2% haematocrit and 50 ml volume according to standard culture protocols [77]. Once a good growth rate of at least 2–3 fold increase after every cycle was ascertained for each parasite line, the culture was synchronized with sorbitol at each subsequent cycle and diluted with O+ red blood cells until 10 such flasks were generated and parasitaemia of 8–10% achieved in each flask. Thereafter, 1 ml infected RBC was sampled at the start of an erythrocytic cycle by centrifuging the contents of one flask in a 15 ml centrifuge tube at 1500 g, and every 12 hrs throughout the 48 hr-cycle. For each sample, once centrifuging was complete, the supernatant was removed from the centrifuge tube and the resulting pellet dissolved in 5 ml of Trizol LS reagent (Invitrogen) then kept at −80°C. This sampling experiment was repeated thrice on different days both V1S and V1S_LM_ to generate 3 biological replicates.

### RNA and DNA extraction

RNA was extracted with phenol-chloroform according to a published protocol [78]. Genomic DNA was extracted from saponin-lysed parasite pellets using a commercial genomic DNA extraction and purification Kit (Qiagen).

### Microarray analysis

All microarray experiments were performed at the Wellcome Trust Sanger Institute, Cambridge, UK using the PFSANGER high density custom tiling-like arrays and following the Sanger Institute Affymetrix protocol [34]. PFSANGER Affymetrix arrays are 25-mer custom designed, covering both strands of *P. falciparum* 3D7 strain, coding and non-coding sequences, for a total of ∼2.5 million probes specific for *P. falciparum*. A total of 24 RNA samples generated from 3 biological replicates of synchronized V1S and V1S_LM_ parasites in culture at 4 time points (0 h, 12 h, 24 h and 36 h) were treated with Turbo DNAse enzyme (Ambion®) according to manufacturer's instructions to remove any contaminating DNA. RNA samples were then quantified and analyzed for quality using the Agilent RNA 6000 Nano Kit (Agilent Technologies). Once the integrity of the RNA was ascertained, double stranded cDNA was synthesized from 8 µg of each RNA sample according to the Affymetrix Eukaryotic gene expression protocol. cDNA was then used as a template in an *in vitro* transcription reaction (IVT) to produce amplified amounts of biotin-labelled complementary RNA (cRNA). 20 ug of cRNA from each time point was fragmented to 25–200 bp fragments after which 15 ug of fragmented cRNA was hybridized onto a PFSANGER microarray as recommended by Affymetrix. Hybridization, washing, staining and scanning was as recommended by the manufacturer. The hybridization intensity for each 25 bp feature from each scan was computed using the Affymetrix software suite. The microarray data was then preprocessed with Bioconductor software and R (www.r-project.org). The arrays were RMA background subtracted, quantile normalized, median polished according to methods previously described [79], and summarized according to the June 2007 release of *P. falciparum* genome annotation, representing a total of 5363 genes. Microarray data were submitted to ArrayExpress under the accession number E-TABM-1101.

### Data mining

An average of 1.5 fold differences in expression of V1S_LM_ relative to V1S was applied as a cut off point for up or down regulation after fitting a linear model to the microarray data (limma package [80]). The cut-off for statistical significance was set at p<0.05. GO enrichment analysis was performed using the GOstats Bioconductor package ([81]). In addition supplemental EDGE analysis [37] was done to identify additional and potentially useful transporter genes. For the segmentation analysis of chromosomes 2 and 10 deletions SnoopCGH software [82] was used with Smith–Waterman algorithm implementation at P<0.01.

### cDNA synthesis

RNA used for cDNA synthesis was obtained from the same V1S (control) and V1S_LM_ (we used the same RNA for RT-qPCR that was used for microarray analysis). For each sample, 5 ug of RNA were used to synthesize cDNA using OligodT_16_ and Bioscript™ reverse transcriptase according to the manaufacturer's instructions.

### Selection of candidate transporters for confirmation by qRT-PCR

PF10_0210 was chosen as control for all qPCR experiments, as its expression did not vary greatly throughout the cycle in both the V1S (control) and V1S_LM_ (drug treated) parasites. Primers were designed based on the mRNA sequences obtained from PlasmoDB (http://www.plasmodb.org/) using primer 3 primer version 0.4.0 primer design software (http://frodo.wi.mit.edu/primer3/) (Table S4). Products were all between 100–150 bp in length, and as much as possible stringent criteria were set to limit self annealing or formation of primer dimmers. qPCR experiments were performed on a Corbett Robotics CAS-1200™ precision liquid handling system (Corbett Life sciences). Each 10 uL amplification reaction was done in 10 uL strips containing 1 uL template, 167 nM/L of forward and reverse primers and 5 uL of SYBR® Green PCR Mastermix (supplied as 2× concentration mix containing SYBR® green I dye, Amplitaq® Gold DNA polymerase, dNTPs with dUTP, passive reference dye and optimized buffer components). Quantitative PCR reactions were done on a Corbett Rotor-gene 6000 thermo-cycler (Corbett Life sciences) and consisted of 95°C for 10 minutes followed by 50 cycles of 95°C for 25 seconds, 58°C for 25 seconds, and 68°c for 30 seconds. Melting analysis of PCR products was done as follows: ramp from 50–99°C rising by 1°C each step, wait 90 seconds of Premelt conditioning on 1^st^ step, and 5 seconds each step afterwards. Corresponding V1S (control) and V1S_LM_ (drug selected) cDNA samples were quantified in the same real time PCR run for each control and candidate gene. For each sample, PCR reactions were done in duplicate. To facilitate relative quantification, a standard curve was generated for each gene by including 4 serial dilutions of 3D7 genomic DNA spanning a wide dynamic range (1∶1, 1∶100, 1∶10000, 1∶1000000) in each run. For the two genes on chromosomes 2 and 10, qPCR experiments were repeated the same way using gDNA as template to estimate gene copy numbers.

For each sample, the following formula was used to calculate relative expression (fold change, FC) between V1S (control) and V1S_LM_ (drug selected):
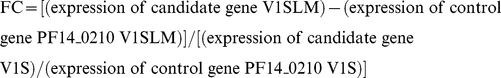



## Supporting Information

Figure S1 **The intraerythrocytic developmental cycle rates of V1S and V1S_LM_.** The maximum likelihood estimates (MLEs) and 95% confidence intervals of the samples are shown. Samples are ordered in the y dimension according to the MLE of hours post invasion (HPI). The MLEs of V1S and V1SLM samples are shown to be comparable across the time course.
(PDF)

Figure S2 **Quality control analyses of the RNA samples and microarray datasets (24 samples).** **A.** Agilent Bioanalyser electrophoresis file run summary of total RNAs. 3 samples were degraded (blue arrows) and not hybridised on PFSANGER arrays. **B.** Boxplot analysis of the raw data following hybridisation. **C.** RNA degradation plot. **D.** Principal Component Analysis plot of the 21 samples prior to remove outliers (indicated by red arrows). Plots were generated in R using the “affycoretools” package.
(PDF)

Table S1 **All genes with F adjusted P<0.05 (589 genes), average expression AveExpr>log2(4) (371 genes), fold change of at least 1.5× (−0.58<log2Ratio<0.58; 266 genes) and with a Bayesian B>0 in at least one time point (192) are listed.** ID = *Plasmodium falciparum* gene ID. log2Ratio = log base 2 of fold change ratio between V1SLM and V1S. B = log odds of DE. AveExp = average expression of a given gene across the whole experiment. F.adj.P.Val = P values adjusted for multiple testing. Product description, TM (number of transmembrane domains), SP (presence of a signal peptide) and GO annotations were taken from www.plasmodb.org.
(PDF)

Table S2 **Known and putative transporters identified by microarray analyses to be differentially expressed during LM transient drug resistance acquisition.** TM, transmembrane domains; SP signal peptide; AP apicoplast targeting signal; qP verified independently by qPCR; RNAseq optimal time of expression as determined by RNAseq [38]; linear modeling for each of the 4 time points as pair-wise comparisons (with log2 Ratio (L2R) and B value), EDGE *p* value, GO cellular component annotation; ref reference.
(PDF)

Table S3 **qPCR analysis of PF10_0017 and PFB0105c using genomic DNA and cDNA templates from V1S_LM_ and V1S** ***P. falciparum*** **isolates.** cDNA samples were taken at the 36 h time point. All data were normalised to PF10_0210.
(DOCX)

Table S4 **List of primers used for RT-qPCR and qPCR assays.**
(DOCX)
