# Supplementary figures and images for: Genome Wide Adaptations of Plasmodium falciparum in Response to Lumefantrine Selective Drug Pressure
Source: PLoS One. 2012 Feb 27;7(2):e31623. doi: 10.1371/journal.pone.0031623 (PMC3288012; doi:10.1371/journal.pone.0031623)

# Maximum Likelihood Estimates V1S and V1SLM Timecourse

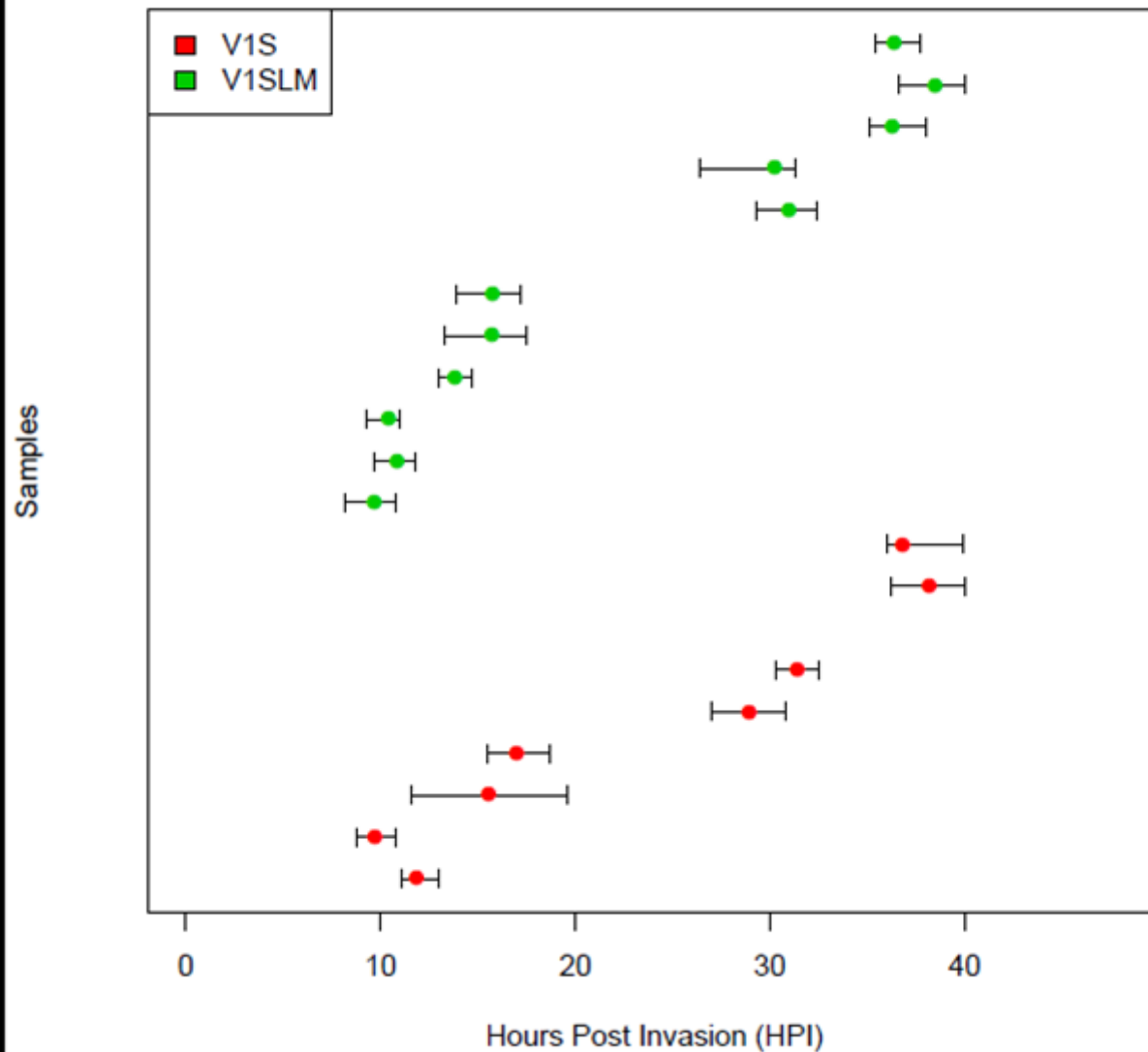

Supplement: Figure S1 — The intraerythrocytic developmental cycle rates of V1S and V1SLM. The maximum likelihood estimates (MLEs) and 95% confidence intervals of the samples are shown. Samples are ordered in the y dimension according to the MLE of hours post invasion (HPI). The MLEs of V1S and V1SLM samples are shown to be comparable across the time course. (PDF) [file pone.0031623.s001.pdf]
