# Supplementary material for: Genome Wide Adaptations of Plasmodium falciparum in Response to Lumefantrine Selective Drug Pressure
Source: PLoS One. 2012 Feb 27;7(2):e31623. doi: 10.1371/journal.pone.0031623 (PMC3288012; doi:10.1371/journal.pone.0031623)
Supplement: Figure S2 — Quality control analyses of the RNA samples and microarray datasets (24 samples). A. Agilent Bioanalyser electrophoresis file run summary of total RNAs. 3 samples were degraded (blue arrows) and not hybridised on PFSANGER arrays. B. Boxplot analysis of the raw data following hybridisation. C. RNA degradation plot. D. Principal Component Analysis plot of the 21 samples prior to remove outliers (indicated by red arrows). Plots were generated in R using the “affycoretools” package. (PDF) [file pone.0031623.s002.pdf]

A

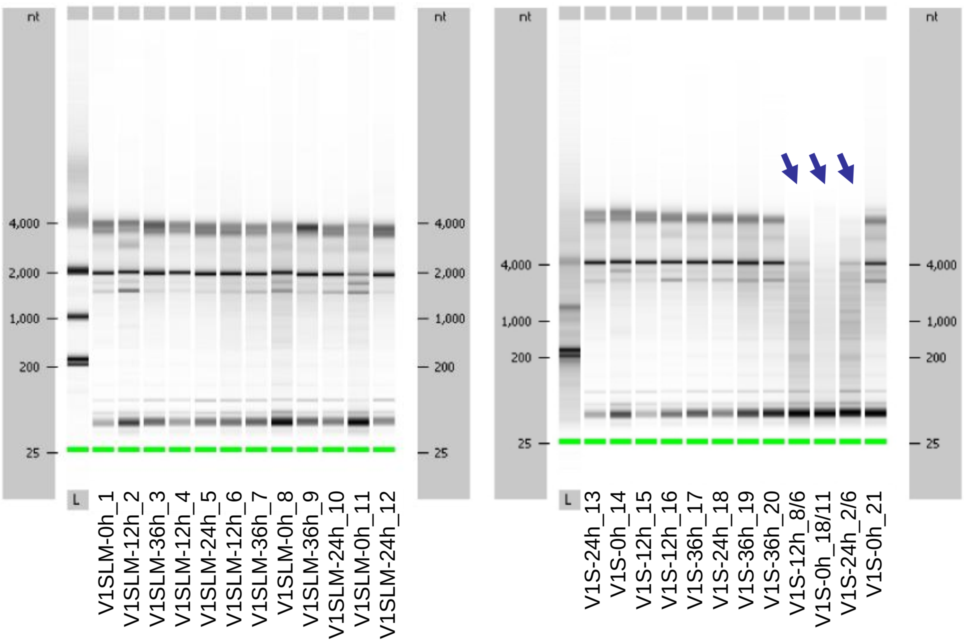
nt
4,000
2,000
1,000
200
25
L
V1SLM-0h_1
V1SLM-12h_2
V1SLM-36h_3
V1SLM-12h_4
V1SLM-24h_5
V1SLM-12h_6
V1SLM-36h_7
V1SLM-0h_8
V1SLM-36h_9
V1SLM-24h_10
V1SLM-0h_11
V1SLM-24h_12
V1S-24h_13
V1S-0h_14
V1S-12h_15
V1S-12h_16
V1S-36h_17
V1S-24h_18
V1S-36h_19
V1S-36h_20
V1S-12h_8/6
V1S-0h_18/11
V1S-24h_2/6
V1S-0h_21

B

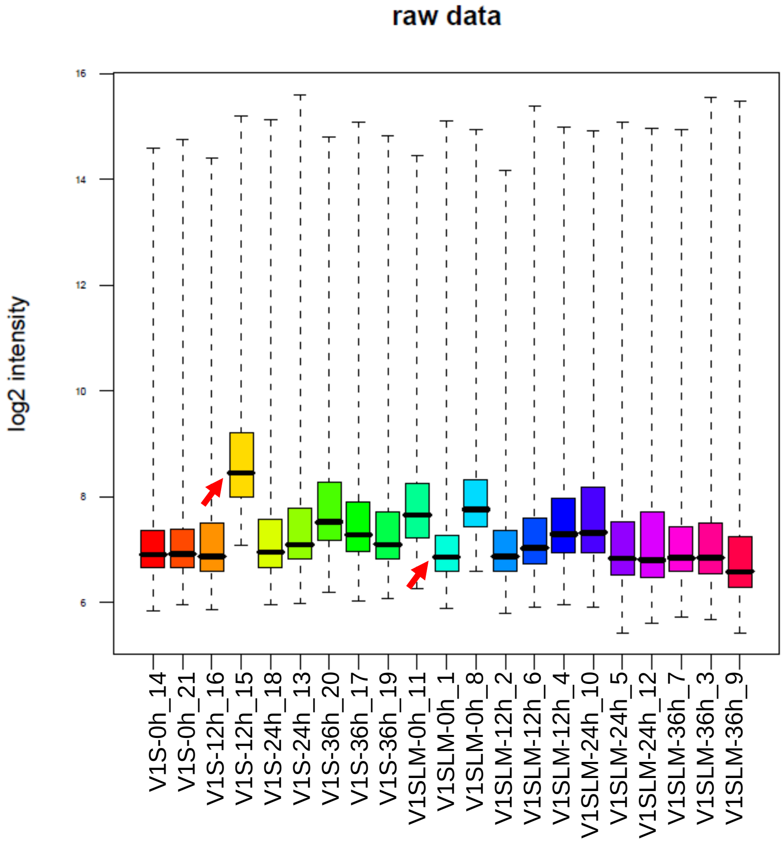
raw data
log2 intensity
16
14
12
10
8
6
V1S-0h_14
V1S-0h_21
V1S-12h_16
V1S-12h_15
V1S-24h_18
V1S-24h_13
V1S-36h_20
V1S-36h_17
V1S-36h_19
V1SLM-0h_11
V1SLM-0h_1
V1SLM-0h_8
V1SLM-12h_2
V1SLM-12h_6
V1SLM-12h_4
V1SLM-24h_10
V1SLM-24h_5
V1SLM-24h_12
V1SLM-36h_7
V1SLM-36h_3
V1SLM-36h_9

C

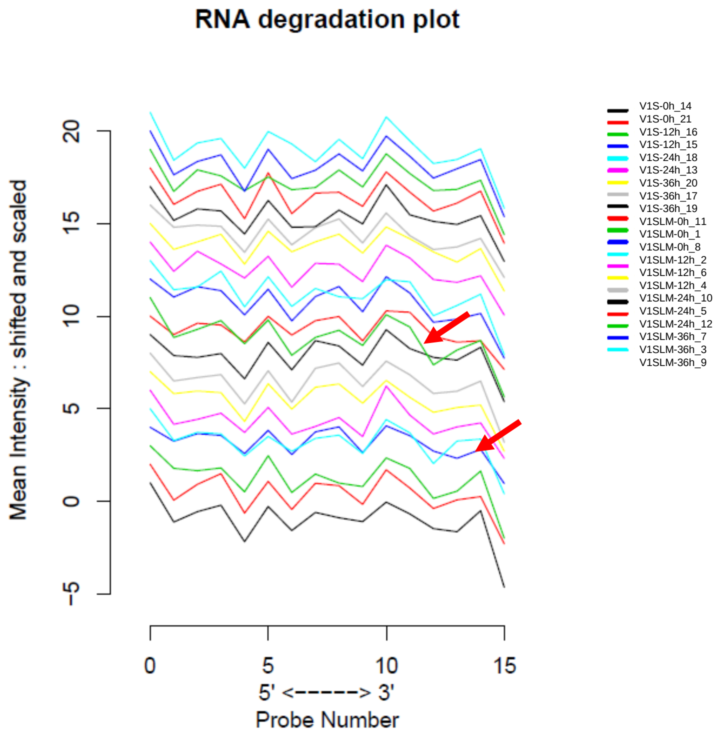

D

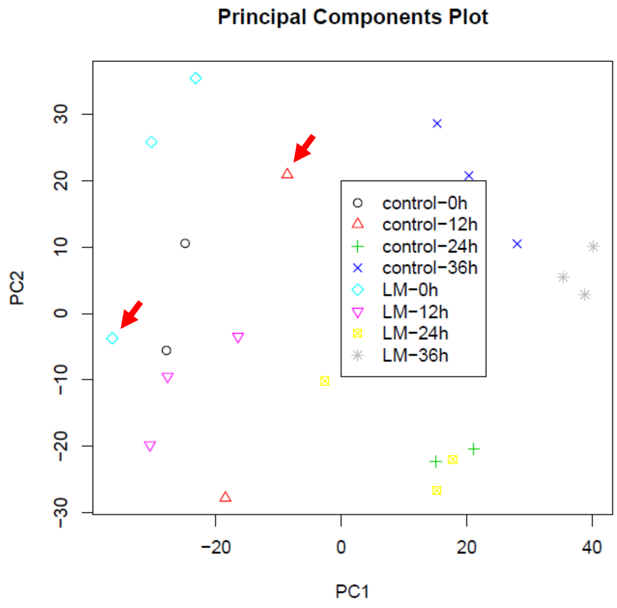

Fig. S2: Quality control analysis of the initial microarray dataset (24 samples). **A**) Agilent Bioanalyser electrophoresis file run summary of total RNAs. 3 samples were degraded (blue arrows) and not hybridised on PFSANGER arrays. **B**) Boxplot analysis of the raw data post hybridisation. **C**) RNA degradation plot and **D**) PCA plot of the 21 samples prior to remove outliers (indicated by red arrows). Plots were generated in R using the affycoretools package.
