# Supplementary material for: Genome Wide Adaptations of Plasmodium falciparum in Response to Lumefantrine Selective Drug Pressure
Source: PLoS One. 2012 Feb 27;7(2):e31623. doi: 10.1371/journal.pone.0031623 (PMC3288012; doi:10.1371/journal.pone.0031623)
Supplement: Table S1 — All genes with F adjusted P<0.05 (589 genes), average expression AveExpr>log2(4) (371 genes), fold change of at least 1.5× (−0.58<log2Ratio<0.58; 266 genes) and with a Bayesian B>0 in at least one time point (192) are listed. ID = Plasmodium falciparum gene ID. log2Ratio = log base 2 of fold change ratio between V1SLM and V1S. B = log odds of DE. AveExp = average expression of a given gene across the whole experiment. F.adj.P.Val = P values adjusted for multiple testing. Product description, TM (number of transmembrane domains), SP (presence of a signal peptide) and GO annotations were taken from www.plasmodb.org. (PDF) [file pone.0031623.s003.pdf]

Table S1: All genes with F adjusted P<0.05 (589 genes), an average expression AveExpr>log2(4) (371 genes), with a fold change of at least 1.5x (-0.58<log2Ratio<0.58; 266 genes) and with a Bayesian B>0 in at least one time point (184) are listed below. ID = *Plasmodium falciparum* gene ID. log2Ratio = log base 2 of fold change ratio between $V1S_{LM}$ and V1S. B = log odds of DE. AveExp = average expression of a given gene across the whole experiment. F.adj.P.Val = P values adjusted for multiple testing. Product description, TM (number of transmembrane domains), SP (presence of a signal peptide) and GO annotations were taken from www.plasmodb.org

	$V1S_{LM}$-V1S 0h		$V1S_{LM}$-V1S 12h		$V1S_{LM}$-V1S 24h		$V1S_{LM}$-V1S 36h									
**ID**	**log2Ratio**	**B**	**log2Ratio**	**B**	**log2Ratio**	**B**	**log2Ratio**	**B**	**AveExpr**	**F. adj.P.Val**	**Product Description**	**TM**	**SP**	**GO Molecular Function**	**GO Biological Process**	**GO Cellular Component**
MAL13P1.190	-0.97	0.39	-0.10	-5.90	-0.05	-5.88	0.42	-3.70	7.53	0.032	proteasome regulatory component, putative	0	null	enzyme regulator activity, endopeptidase activity	regulation of protein catabolic process, ubiquitin-dependent protein catabolic process	proteasome complex, proteasome regulatory particle
MAL13P1.21	0.12	-6.10	-0.24	-4.93	0.05	-5.84	0.63	1.55	4.62	0.028	conserved Plasmodium protein, unknown function	0	null	null	null	null
MAL13P1.235	-1.35	2.09	-0.12	-5.88	-0.18	-5.57	-0.22	-5.76	7.93	0.025	conserved Plasmodium membrane protein, unknown function	4	null	null	null	null
MAL13P1.257	-0.66	0.98	-0.22	-4.95	-0.06	-5.79	-0.19	-4.88	4.20	0.029	conserved Plasmodium protein, unknown function	0	null	null	null	null
MAL13P1.261	-0.75	1.05	-0.10	-5.82	-0.14	-5.35	0.09	-6.08	4.65	0.033	conserved Plasmodium protein, unknown function	0	null	null	null	null
MAL13P1.265	-0.71	0.35	-0.44	-3.31	-0.35	-3.29	-0.03	-6.39	5.17	0.025	conserved Plasmodium protein, unknown function	0	null	null	null	null
MAL13P1.271	-0.85	1.49	-0.28	-4.90	0.08	-5.77	0.32	-3.70	10.38	0.023	V-type ATPase, putative	4	yes	proton-transporting ATPase activity, rotational mechanism, ATP binding	ATP metabolic process, proton transport	vacuolar proton-transporting V-type ATPase complex, integral to membrane
MAL13P1.283	-0.63	1.90	-0.05	-5.92	0.12	-5.25	-0.32	-1.88	5.78	0.019	TCP-1/cpn60 chaperonin family, putative	0	null	ATPase activity, coupled, unfolded protein binding, ATP binding	protein folding	chaperonin-containing T-complex
MAL13P1.300	-0.75	1.73	-0.27	-4.63	-0.25	-4.09	0.08	-6.15	5.47	0.023	conserved Plasmodium protein, unknown function	3	yes	null	null	apicoplast
MAL13P1.345	-0.58	2.52	-0.14	-5.21	-0.13	-4.83	-0.15	-4.63	5.04	0.020	conserved Plasmodium protein, unknown function	0	null	null	null	null
MAL13P1.356	0.67	0.82	0.36	-3.74	0.12	-5.42	-0.24	-4.29	4.16	0.025	erythrocyte membrane protein 1, PfEMP1	0	null	receptor activity	pathogenesis	integral to membrane
MAL13P1.76	-0.61	2.76	-0.17	-4.92	0.09	-5.39	-0.17	-4.40	4.50	0.018	TFIIH basal transcription factor subunit	0	null	RNA polymerase II transcription factor activity, zinc ion binding, protein binding	DNA repair, regulation of transcription	nucleus
MAL13P1.79	-0.80	1.79	-0.02	-5.97	0.10	-5.65	0.27	-4.02	7.90	0.024	conserved Plasmodium protein, unknown function	0	null	null	null	null
MAL13P1.8	1.57	1.72	0.50	-4.90	0.06	-5.89	-0.94	-0.79	5.52	0.016	RIF pseudogene	1	null	null	null	null
MAL13P1.82	-0.87	0.93	-0.62	-2.32	-0.25	-4.70	0.29	-4.48	5.72	0.021	phosphatidylinositol synthase	3	yes	CDP-diacylglycerol-inositol 3-phosphatidyltransferase activity	phosphatidylinositol biosynthetic process	membrane
MAL13P1.93	-0.92	2.05	-0.02	-5.97	-0.06	-5.84	-0.20	-5.26	4.25	0.025	conserved Plasmodium protein, unknown function	0	null	null	null	null
MAL7P1.139	-0.90	3.81	-0.05	-5.91	0.00	-5.92	0.08	-6.10	5.15	0.017	mago nashi protein homolog, putative	0	null	null	sex determination	nucleus
MAL7P1.174	1.37	-3.00	3.11	1.85	2.66	2.41	1.57	-0.81	6.59	0.003	Plasmodium exported protein (PHISTb), unknown function	1	null	null	null	null
MAL7P1.175	0.68	-1.76	1.21	1.55	0.79	0.06	0.18	-5.75	4.23	0.011	Serine/Threonine protein kinase, FIKK family, pseudogene	0	null	protein serine/threonine kinase activity, ATP binding	protein amino acid phosphorylation	null
MAL7P1.300	-1.67	3.04	0.29	-5.53	-0.15	-5.71	-0.98	0.05	7.49	0.011	40S ribosomal protein S29, putative	0	null	structural constituent of ribosome	translation	ribosome
MAL7P1.56	0.65	0.06	0.24	-4.95	0.10	-5.61	-0.27	-4.02	4.01	0.032	erythrocyte membrane protein 1, PfEMP1	0	null	cell adhesion molecule binding, receptor activity	pathogenesis, antigenic variation, cell-cell adhesion, cytoadherence to microvasculature, mediated by parasite protein, rosetting	infected host cell surface knob, host cell plasma membrane, integral to membrane
MAL7P1.64	-0.70	3.26	-0.30	-3.48	-0.30	-2.38	-0.34	-1.26	4.13	0.008	serpentine receptor, putative, PfSR25	8	yes	null	null	null
MAL8P1.107	-1.00	2.29	-0.51	-3.18	-0.18	-5.23	-0.20	-5.42	4.98	0.019	conserved Plasmodium protein, unknown function	1	null	null	null	null
MAL8P1.142	-1.36	2.27	-0.15	-5.83	-0.22	-5.38	0.29	-5.26	9.65	0.023	proteasome beta-subunit	0	null	threonine-type endopeptidase activity, endopeptidase activity	ubiquitin-dependent protein catabolic process	proteasome core complex
MAL8P1.150	0.13	-6.26	0.07	-5.94	-0.23	-5.26	0.94	1.33	4.63	0.031	conserved Plasmodium protein, unknown function	0	null	phosphorus-oxygen lyase activity	intracellular signaling cascade, cyclic nucleotide biosynthetic process	null
MAL8P1.220	1.10	2.05	0.43	-4.27	0.18	-5.38	-0.42	-3.32	4.32	0.019	erythrocyte membrane protein 1, PfEMP1	0	null	receptor activity	pathogenesis	integral to membrane
MAL8P1.36	-1.14	6.70	-0.22	-4.92	-0.11	-5.49	-0.16	-5.14	5.71	0.004	conserved Plasmodium protein, unknown function	1	null	null	null	null
MAL8P1.82	0.16	-5.86	0.00	-5.98	0.04	-5.88	0.69	1.88	5.55	0.027	Vacuolar sorting protein VPS9, putative	0	null	null	null	null
MAL8P1.9	-0.76	1.99	0.10	-5.77	-0.06	-5.77	-0.44	-0.82	6.30	0.016	u6 snRNA-associated Sm-like protein, putative	0	null	U6 snRNA binding	nuclear mRNA splicing, via spliceosome	small nucleolar ribonucleoprotein complex
PF07_0013	-1.21	2.96	-0.22	-5.48	0.08	-5.80	-0.11	-6.15	4.50	0.021	conserved Plasmodium protein, unknown function	0	null	null	null	null
PF07_0048	1.11	2.06	0.42	-4.37	0.16	-5.49	-0.40	-3.67	4.24	0.020	erythrocyte membrane protein 1, PfEMP1	0	null	receptor activity, cell adhesion molecule binding	cell-cell adhesion, pathogenesis, cytoadherence to microvasculature, mediated by parasite protein, rosetting, antigenic variation	host cell plasma membrane, infected host cell surface knob, integral to membrane
PF07_0072	-0.14	-6.22	0.29	-5.25	-0.15	-5.61	0.91	1.49	5.26	0.029	calcium-dependent protein kinase 4	0	null	calcium-dependent protein serine/threonine phosphatase activity, protein tyrosine kinase activity, ATP binding, protein serine/threonine kinase activity, calcium ion binding	protein amino acid phosphorylation	null
PF07_0088	-1.06	1.68	0.09	-5.90	0.17	-5.46	-0.36	-4.09	7.63	0.024	40S ribosomal protein S5, putative	0	null	structural constituent of ribosome	translation	cytosolic small ribosomal subunit
PF08_0031	-1.49	0.01	-0.92	-3.50	0.11	-5.85	0.62	-4.04	6.58	0.028	oxoglutarate/malate translocator protein, putative	1	null	dicarboxylic acid transmembrane transporter activity, binding, oxoglutarate:malate antiporter activity	dicarboxylic acid transport, mitochondrial transport, malate transport, alpha-ketoglutarate transport	mitochondrial inner membrane
PF08_0053	-1.04	1.31	-0.30	-5.16	-0.05	-5.88	-0.33	-4.47	4.77	0.026	BRIX domain, putative	0	null	null	null	null
PF08_0058	0.38	-4.54	0.16	-5.71	-0.10	-5.76	0.94	2.83	4.67	0.020	MAC/Perforin, putative	0	null	null	null	null
PF08_0071	-1.16	0.08	-0.64	-3.90	-0.12	-5.78	0.13	-6.19	9.44	0.037	Fe-superoxide dismutase	0	null	superoxide dismutase activity, metal ion binding	response to oxidative stress, superoxide metabolic process	null
PF08_0082	0.23	-5.68	0.30	-5.05	-0.30	-4.52	1.06	3.82	4.22	0.013	conserved Plasmodium protein, unknown function	0	null	null	null	null

PF08_0110	-1.04	3.98	-0.08	-5.87	-0.25	-4.41	-0.15	-5.65	6.48	0.013	PfRab18, GTPase	0	null	protein binding, GTP binding, GTPase activity	nucleocytoplasmic transport, small GTPase mediated signal transduction, intracellular protein transport	intracellular
PF08_0124	-0.64	6.19	-0.13	-4.86	0.01	-5.91	-0.20	-2.17	5.06	0.004	conserved Plasmodium protein, unknown function	0	null	null	null	null
PF10_0004	1.46	2.86	0.45	-4.70	0.23	-5.37	-0.58	-2.67	4.52	0.015	rifin	1	null	molecular_function	antigenic variation	host cell plasma membrane, membrane
PF10_0005	0.71	2.00	0.22	-4.83	0.07	-5.74	-0.30	-2.96	4.24	0.020	rifin	1	null	molecular_function	antigenic variation	membrane, host cell plasma membrane
PF10_0015	2.46	4.46	1.22	-2.24	0.82	-3.05	1.64	2.50	4.55	0.002	acyl CoA binding protein, isoform 1, ACBP1	0	null	acyl-CoA binding	fatty acid metabolic process	cellular_component
PF10_0016	1.95	-3.55	5.84	3.26	4.75	3.39	4.08	3.51	8.07	0.000	acyl CoA binding protein, isoform 2, ACBP2	0	null	acyl-CoA binding	fatty acid metabolic process	cellular_component
PF10_0017	0.69	-3.21	0.83	-2.74	2.95	8.01	0.76	-1.39	4.29	0.000	Plasmodium exported protein (PHISTa), unknown function	1	null	molecular_function	biological_process	membrane
PF10_0019	2.47	-0.77	5.72	4.91	4.45	4.60	3.36	3.72	9.04	0.000	early transcribed membrane protein 10.1, etramp 10.1	2	yes	molecular_function	biological_process	cellular_component, membrane
PF10_0020	0.16	-6.20	0.32	-5.29	1.96	5.65	2.01	8.15	4.48	0.000	alpha/beta hydrolase, putative	0	null	null	null	null
PF10_0029	-0.35	-3.88	0.10	-5.81	0.71	1.41	0.00	-6.41	4.64	0.025	conserved Plasmodium protein, unknown function	0	null	thiol oxidase activity	oxidation reduction	null
PF10_0094	0.13	-6.37	0.09	-5.95	-0.27	-5.46	1.37	1.48	4.55	0.031	tubulin-tyrosine ligase, putative	0	null	tubulin-tyrosine ligase activity	protein modification process	null
PF10_0104	-0.69	1.06	-0.11	-5.73	0.01	-5.91	0.04	-6.36	4.72	0.036	conserved Plasmodium protein, unknown function	0	yes	null	null	membrane
PF10_0127	-0.58	1.75	-0.04	-5.93	-0.14	-4.87	-0.10	-5.68	4.50	0.025	conserved Plasmodium protein, unknown function	1	yes	protein binding, molecular_function, zinc ion binding	biological_process	membrane
PF10_0160	-0.87	1.53	-0.22	-5.28	0.07	-5.80	-0.28	-4.38	6.14	0.025	Serine/Threonine protein kinase, FIKK family	1	null	protein serine/threonine kinase activity, ATP binding, molecular_function	biological_process, protein amino acid phosphorylation	membrane, mitochondrion, cellular_component
PF10_0180	-0.75	1.14	-0.21	-5.24	-0.07	-5.76	-0.08	-6.20	7.31	0.032	conserved Plasmodium protein, unknown function	0	null	null	null	null
PF10_0181	-1.06	2.46	0.05	-5.95	-0.07	-5.83	-0.13	-5.97	4.19	0.024	conserved protein, unknown function	1	null	molecular_function	biological_process	membrane
PF10_0193	0.36	-4.85	0.17	-5.70	0.47	-3.14	1.26	5.19	5.02	0.007	microtubule-associated protein 1 light chain 3, putative	0	null	molecular_function	biological_process	cellular_component
PF10_0264	-0.91	0.01	-0.10	-5.88	-0.03	-5.90	-0.50	-2.70	7.86	0.031	40S ribosomal protein S2B, putative	0	null	structural constituent of ribosome	translation	cytosolic small ribosomal subunit
PF10_0266	-0.89	3.16	-0.37	-3.65	-0.14	-5.29	-0.05	-6.31	4.73	0.017	small subunit rRNA processing stabilizing factor, putative	0	null	null	null	null
PF10_0323	-1.45	0.31	-0.03	-5.98	-0.51	-4.44	0.04	-6.40	12.70	0.040	early transcribed membrane protein 10.2, etramp 10.2	2	yes	molecular_function	biological_process	membrane
PF10_0366	-0.99	0.98	-0.45	-4.21	-0.04	-5.89	-0.48	-2.74	9.73	0.023	ADP/ATP transporter on adenylate translocase	3	null	ATP:ADP antiporter activity, binding	transport	mitochondrial inner membrane
PF10_0401	1.16	1.59	0.37	-4.90	-0.12	-5.71	-0.06	-6.35	4.19	0.028	rifin	1	yes	molecular_function	antigenic variation	membrane, host cell plasma membrane
PF10_0406	0.96	0.36	0.51	-3.91	0.26	-5.00	-0.41	-3.77	4.07	0.025	erythrocyte membrane protein 1, PfEMP1	0	null	host cell surface receptor binding, receptor activity, cell adhesion molecule binding	antigenic variation, rosetting, cytoadherence to microvasculature, mediated by parasite protein, pathogenesis, cell-cell adhesion	integral to membrane, host cell plasma membrane, infected host cell surface knob
PF10_0415	-0.80	1.49	-0.13	-5.68	-0.22	-4.74	0.10	-6.06	4.73	0.027	conserved Plasmodium protein, unknown function	1	null	null	null	null
PF11_0021	1.57	1.53	0.69	-4.13	0.23	-5.55	-0.66	-3.20	4.32	0.021	rifin	2	yes	molecular_function	antigenic variation	host cell plasma membrane, membrane
PF11_0043	-0.60	0.30	-0.29	-4.26	-0.08	-5.68	-0.02	-6.40	4.47	0.037	60S ribosomal protein P1, putative	0	null	structural constituent of ribosome	translation, translational elongation	large ribosomal subunit, ribosome
PF11_0051	-0.66	2.06	-0.31	-3.65	0.01	-5.91	0.02	-6.38	6.26	0.023	phenylalanyl-tRNA synthetase beta chain, putative	0	null	ATP binding, RNA binding, phenylalanine-tRNA ligase activity	phenylalanyl-tRNA aminoacylation	phenylalanine-tRNA ligase complex, cytoplasm
PF11_0172	-0.63	-2.96	-0.16	-5.76	0.51	-3.19	0.84	0.44	7.51	0.023	folate/biopterin transporter, putative	11	null	molecular_function	biological_process	cellular_component
PF11_0309	-1.03	3.62	0.24	-5.09	-0.24	-4.53	-0.09	-6.11	4.72	0.015	conserved Plasmodium protein, unknown function	2	null	molecular_function	biological_process	membrane
PF11_0400	-0.66	0.27	-0.04	-5.94	-0.16	-5.20	-0.10	-6.03	5.35	0.044	conserved Plasmodium protein, unknown function	0	null	molecular_function	biological_process	membrane
PF11_0447	-0.66	0.44	-0.12	-5.68	-0.07	-5.76	0.05	-6.30	5.67	0.044	translation initiation factor eIF-1A, putative	0	null	translation initiation factor activity, RNA binding	translational initiation	eukaryotic 43S preinitiation complex
PF11_0458	-0.62	0.07	0.08	-5.85	-0.04	-5.87	-0.18	-5.14	4.10	0.044	conserved Plasmodium protein, unknown function	0	null	catalytic activity, RNA polymerase II transcription factor activity	transcription initiation from RNA polymerase II promoter	null
PF11_0464	0.14	-5.98	0.01	-5.98	-0.25	-4.43	0.59	0.51	4.37	0.036	Ser/Thr protein kinase, putative	0	null	ATP binding, protein kinase activity, protein tyrosine kinase activity, protein serine/threonine kinase activity	protein amino acid phosphorylation, biological_process	apicoplast, membrane
PF13_0006	1.14	1.37	0.56	-3.78	0.24	-5.17	-0.38	-4.30	4.32	0.023	rifin	1	yes	molecular_function	antigenic variation	host cell plasma membrane, membrane
PF13_0049	-0.68	1.16	0.19	-5.18	0.08	-5.66	0.09	-6.03	5.48	0.031	60S ribosomal protein L24, putative	0	null	structural constituent of ribosome	translation	cytosolic large ribosomal subunit
PF13_0051	-0.58	0.92	-0.20	-4.94	-0.13	-5.23	-0.22	-3.96	4.90	0.025	snornp protein gar1 homologue, putative	0	null	rRNA binding	rRNA processing, RNA modification	small nucleolar ribonucleoprotein complex
PF13_0128	-2.01	6.13	0.18	-5.74	-0.01	-5.91	-0.27	-5.37	5.43	0.006	beta-hydroxyacyl-ACP dehydratase precursor	1	yes	catalytic activity, (3R)-hydroxymyristoyl-[acyl-carrier-protein] dehydratase activity	fatty acid biosynthetic process	apicoplast
PF13_0214	-0.86	3.44	-0.19	-5.19	0.00	-5.91	-0.23	-4.34	7.47	0.016	elongation factor 1-gamma, putative	0	null	translation elongation factor	translational elongation	eukaryotic translation elongation factor 1
PF13_0282	-0.71	0.53	-0.47	-2.92	-0.27	-4.18	0.06	-6.30	4.62	0.025	proteasome subunit, putative	0	null	endopeptidase activity, threonine-type endopeptidase activity	ubiquitin-dependent protein catabolic process	proteasome core complex
PF13_0285	-0.07	-6.32	0.15	-5.55	0.07	-5.74	0.69	2.78	5.96	0.023	inositol-polyphosphate 5-phosphatase	3	null	inositol-polyphosphate 5-phosphatase activity	null	null
PF13_0300	-0.94	0.27	-0.43	-4.43	0.23	-5.15	0.00	-6.41	5.83	0.036	mitochondrial inner membrane translocase, putative	2	null	protein transporter activity	protein targeting to mitochondrion	mitochondrial inner membrane presequence translocase complex, mitochondrial inner membrane

PF13_0305	-1.31	4.99	-0.56	-2.78	-0.32	-4.01	-0.27	-4.68	5.76	0.006	elongation factor-1 alpha	0	null	GTP binding, GTPase activity, translation elongation factor activity	translational elongation	eukaryotic translation elongation factor 1 complex
PF13_0310	-0.61	0.59	-0.16	-5.37	-0.02	-5.90	0.07	-6.16	4.91	0.040	preribosomal processosome UTP, putative	0	null	null	null	null
PF13_0358	-1.92	1.99	-0.40	-5.47	0.23	-5.64	-0.22	-6.08	6.11	0.025	mitochondrial import inner membrane translocase, putative	0	null	P-P-bond-hydrolysis-driven protein transmembrane transporter activity	protein import into mitochondrial inner membrane, protein targeting to mitochondrion	mitochondrial intermembrane space protein transporter complex, mitochondrial intermembrane space
PF14_0003	0.97	0.98	0.42	-4.37	0.05	-5.87	-0.10	-6.18	4.31	0.031	rifin	3	yes	molecular_function	antigenic variation	membrane, host cell plasma membrane
PF14_0020	-1.42	1.96	-0.14	-5.86	0.04	-5.90	0.52	-3.59	7.68	0.023	choline kinase	0	null	choline kinase activity	lipid metabolic process	cellular_component
PF14_0071	-0.65	0.65	-0.11	-5.69	-0.15	-5.17	0.15	-5.41	4.48	0.033	conserved Plasmodium protein, unknown function	0	null	molecular_function	biological_process	apicoplast
PF14_0078	-0.78	2.50	-0.18	-5.31	0.01	-5.91	-0.38	-1.71	6.49	0.016	HAP protein	1	null	aspartic-type endopeptidase activity	proteolysis, biological_process	membrane
PF14_0143	-0.03	-6.44	0.07	-5.86	-0.04	-5.85	0.63	2.07	5.19	0.027	Atypical protein kinase, ABC-1 family, putative	0	null	null	null	null
PF14_0191	-0.85	1.66	-0.24	-5.12	-0.15	-5.36	0.14	-5.82	7.68	0.025	conserved Plasmodium protein, unknown function	0	null	null	null	null
PF14_0209	-0.64	0.36	-0.06	-5.90	-0.07	-5.75	0.08	-6.16	4.29	0.046	conserved Plasmodium protein, unknown function	0	null	null	null	null
PF14_0231	-1.00	0.27	-0.10	-5.90	-0.05	-5.88	-0.17	-5.91	5.69	0.047	60S ribosomal protein L7-3, putative	0	null	structural constituent of ribosome	ribosome biogenesis, translation	cytosolic large ribosomal subunit
PF14_0242	-1.11	1.56	-0.27	-5.35	-0.04	-5.90	-0.02	-6.41	5.94	0.030	arginine-N-methyltransferase, putative	0	null	arginine N-methyltransferase activity	biological_process, metabolic process	cellular_component
PF14_0293	0.12	-5.90	0.17	-5.22	-0.17	-4.79	0.65	3.78	4.07	0.015	conserved Plasmodium protein, unknown function	0	yes	null	null	null
PF14_0424	-1.06	1.82	-0.39	-4.50	0.07	-5.83	-0.10	-6.19	4.79	0.025	conserved Plasmodium protein, unknown function	1	null	molecular_function	biological_process	membrane
PF14_0425	-1.15	3.85	-0.32	-4.68	-0.09	-5.75	0.01	-6.41	11.86	0.015	fructose-bisphosphate aldolase	0	null	fructose-bisphosphate aldolase activity	gluconeogenesis, glycolysis	cellular_component
PF14_0429	-0.85	0.19	-0.02	-5.98	-0.04	-5.89	-0.17	-5.72	4.53	0.048	RNA helicase, putative	0	null	ATP-dependent helicase activity, nucleic acid binding, ATP binding, helicase activity	biological_process	cellular_component
PF14_0439	-0.93	0.91	-0.21	-5.50	0.31	-4.42	-0.34	-4.06	8.01	0.025	M17 leucyl aminopeptidase	0	null	manganese ion binding	proteolysis	apicoplast
PF14_0457	-0.41	3.33	-0.11	-4.86	-0.14	-3.35	-0.70	10.58	5.20	0.000	conserved Plasmodium protein, unknown function	0	null	null	null	null
PF14_0466	-0.58	0.97	-0.60	0.13	-0.30	-2.66	0.00	-6.41	4.53	0.013	Appr-1-p processing domain protein	0	null	null	null	null
PF14_0482	-0.80	0.64	-0.10	-5.84	-0.13	-5.55	-0.14	-5.83	6.01	0.038	conserved Plasmodium protein, unknown function	0	null	molecular_function	biological_process	cellular_component
PF14_0495	0.28	-5.70	0.10	-5.91	-0.21	-5.44	1.37	4.27	4.43	0.013	rhoptry neck protein 2	1	yes	molecular_function	biological_process	membrane
PF14_0586	0.08	-6.40	0.09	-5.94	-0.05	-5.89	1.14	1.54	4.59	0.031	conserved Plasmodium protein, unknown function	0	null	null	null	null
PF14_0619	-0.69	1.83	-0.25	-4.62	0.11	-5.48	-0.08	-6.06	4.35	0.025	conserved Plasmodium protein, unknown function	0	null	null	null	null
PF14_0627	-0.73	0.58	-0.17	-5.50	0.04	-5.87	-0.37	-2.84	6.38	0.026	40S ribosomal protein S3, putative	0	null	RNA binding, structural constituent of ribosome	translation	cytosolic small ribosomal subunit
PF14_0670	-0.31	-5.70	0.32	-5.36	-0.08	-5.86	-1.14	1.82	7.24	0.025	conserved Plasmodium protein, unknown function	0	null	null	null	null
PF14_0759	1.31	1.03	0.15	-5.85	0.25	-5.38	-0.82	-1.13	4.33	0.021	conserved Plasmodium protein, unknown function, pseudogene	1	yes	molecular_function	biological_process	membrane
PF14_0770	2.46	1.41	0.55	-5.45	0.37	-5.53	-1.49	-1.01	4.36	0.018	rifin	1	yes	molecular_function	antigenic variation	membrane, host cell plasma membrane
PFA0005w	0.86	0.44	0.20	-5.54	0.04	-5.88	-0.36	-3.81	4.00	0.031	erythrocyte membrane protein 1, PfEMP1	0	null	receptor activity, cell adhesion molecule binding	pathogenesis, cytoadherence to microvasculature, mediated by parasite protein, cell-cell adhesion, antigenic variation, rosetting	infected host cell surface knob, host cell plasma membrane, integral to membrane
PFA0010c	1.78	1.29	0.78	-4.21	0.20	-5.71	-0.65	-3.94	5.25	0.023	rifin	1	null	molecular_function	antigenic variation	membrane, host cell plasma membrane
PFA0050c	1.04	0.33	0.27	-5.41	0.20	-5.43	-0.53	-2.89	4.52	0.027	rifin	2	yes	molecular_function	antigenic variation	membrane, host cell plasma membrane
PFA0095c	0.98	1.76	0.26	-5.19	0.21	-5.13	-0.36	-3.68	4.27	0.022	rifin	1	yes	molecular_function	antigenic variation	host cell plasma membrane, membrane
PFA0230c	-0.66	-3.08	-0.01	-5.98	0.17	-5.60	-1.12	2.37	8.10	0.020	conserved Plasmodium protein, unknown function	0	null	null	null	null
PFA0590w	-0.08	-6.32	-0.33	-4.41	0.67	0.42	0.32	-3.60	5.28	0.027	ABC transporter, (CT family), putative, PfMRP1	11	null	ATPase activity, coupled to transmembrane movement of substances, ATP binding	transport	integral to membrane, membrane
PFA0615w	-0.63	-3.51	-0.20	-5.73	-0.55	-3.33	-4.25	14.26	4.68	0.000	Plasmodium exported protein, unknown function	2	yes	null	null	null
PFA0645c	2.32	1.54	0.22	-5.88	-0.07	-5.90	-1.23	-1.81	5.14	0.021	hypothetical protein	2	null	null	null	null
PFA0735w	-0.39	-5.36	-1.50	0.64	-1.18	0.60	-0.50	-4.06	4.32	0.013	Plasmodium exported protein (PHISTa), unknown function	1	yes	null	null	null
PFB0045c	1.50	2.08	0.56	-4.43	0.28	-5.25	-0.76	-1.75	4.04	0.016	erythrocyte membrane protein 1 (PfEMP1), truncated	2	null	null	null	null
PFB0085c	0.95	2.63	0.25	-5.05	0.04	-5.88	0.60	0.39	4.05	0.011	DNAJ protein, putative	1	null	unfolded protein binding, heat shock protein binding, molecular_function	protein folding, biological_process	host cell part
PFB0090c	1.30	-0.14	1.72	1.00	0.46	-4.55	0.35	-5.35	6.74	0.015	RESA-like protein with PHIST and DnaJ domains	1	yes	heat shock protein binding, unfolded protein binding, molecular_function	protein folding	cellular_component
PFB0100c	0.76	0.00	1.09	1.76	1.21	4.44	0.74	1.55	6.00	0.000	knob-associated histidine-rich protein	0	yes	null	null	host cell part
PFB0105c	0.82	-2.85	1.43	0.04	1.04	-0.58	1.37	2.61	5.25	0.005	Plasmodium exported protein (PHISTc), unknown function	1	null	molecular_function	biological_process	membrane
PFB0190c	-0.03	-6.43	-0.08	-5.86	-0.21	-4.67	0.58	0.86	4.11	0.033	conserved Plasmodium protein, unknown function	0	yes	transferase activity	null	null
PFB0245c	-0.73	0.17	0.11	-5.80	-0.13	-5.52	-0.23	-4.86	5.10	0.039	DNA-directed RNA polymerase II 16 kDa subunit, putative	0	null	nucleotide binding, DNA-directed RNA polymerase activity	transcription from RNA polymerase II promoter	cellular_component
PFB0290c	-1.79	0.76	-0.04	-5.98	-0.17	-5.78	-0.07	-6.39	6.16	0.042	transcription factor, putative	0	null	transcription factor activity, zinc ion binding	transcription from RNA polymerase III promoter, regulation of transcription	DNA-directed RNA polymerase III complex
PFB0445c	-0.70	3.13	0.20	-4.74	0.08	-5.62	-0.14	-5.16	6.10	0.018	DEAD box helicase, UAP56	0	null	nucleic acid binding, ATP binding, ATP-dependent RNA helicase activity	biological_process	cellular_component
PFB0595w	-0.84	0.10	-0.46	-3.92	-0.26	-4.79	-0.29	-4.61	5.82	0.028	heat shock 40 kDa protein, putative	0	null	heat shock protein binding, unfolded protein binding	response to unfolded protein, protein folding, response to heat	cellular_component

PFB0725c	-0.51	-4.22	-0.83	-2.30	-1.16	1.56	-0.40	-4.34	5.72	0.016	zinc finger protein, putative	4	yes	zinc ion binding	biological_process	apicoplast, membrane
PFB1045w	1.48	0.02	0.45	-5.28	0.40	-5.06	-0.08	-6.37	4.13	0.044	erythrocyte membrane protein 1 (PfEMP1), truncated	0	yes	null	null	null
PFC0005w	0.64	1.58	0.18	-5.12	0.07	-5.69	-0.26	-3.42	4.05	0.023	erythrocyte membrane protein 1, PfEMP1	0	null	receptor activity	pathogenesis	integral to membrane
PFC0030c	1.38	0.09	0.28	-5.66	0.25	-5.51	-0.61	-3.73	4.33	0.033	rifin	2	yes	molecular_function	antigenic variation	membrane, host cell plasma membrane
PFC0235w	-0.20	-4.55	0.10	-5.61	-0.10	-5.28	0.58	4.48	4.33	0.011	conserved Plasmodium protein, unknown function	0	null	actin binding, protein binding	cytoskeleton organization, vesicle-mediated transport	membrane
PFC0282w	-0.78	4.29	-0.02	-5.96	-0.10	-5.37	-0.34	-1.27	4.96	0.009	conserved Plasmodium protein, unknown function	2	yes	null	null	null
PFC0340w	0.01	-6.46	-0.82	0.72	-0.04	-5.87	0.05	-6.32	5.93	0.042	DNA polymerase epsilon subunit B, putative	0	null	DNA binding, DNA-directed DNA polymerase activity	DNA replication	delta DNA polymerase complex
PFC0395w	-0.72	1.53	0.14	-5.55	0.23	-4.32	-0.30	-3.17	6.76	0.021	asparagine synthetase, putative	0	null	asparagine synthase (glutamine-hydrolyzing) activity	asparagine biosynthetic process	null
PFC0725c	-0.91	1.14	-0.17	-5.63	0.04	-5.88	-0.02	-6.41	5.93	0.035	formate-nitrate transporter, putative	6	null	transporter activity	transport	membrane
PFC0840w	0.33	-4.92	-0.26	-5.24	0.13	-5.63	0.69	0.12	4.62	0.038	P-type ATPase, putative, PfATPase7	10	null	phospholipid-translocating ATPase activity, ATP binding, ATPase activity, coupled to transmembrane movement of ions, phosphorylative mechanism, magnesium ion binding, transporter activity	phospholipid transport, cation transport	integral to plasma membrane
PFC0900w	-0.72	0.23	-0.38	-3.94	0.01	-5.91	-0.08	-6.20	6.76	0.037	T-complex protein 1 epsilon subunit, putative	0	null	ATP binding, unfolded protein binding	protein folding	chaperonin-containing T-complex
PFC1016w	-0.66	2.24	-0.06	-5.89	-0.07	-5.67	-0.06	-6.20	4.03	0.025	conserved Plasmodium protein, unknown function	0	null	null	null	null
PFC1120c	1.40	1.95	0.52	-4.46	0.20	-5.53	-0.58	-3.03	4.45	0.020	var (3D7-varT3-2)	0	null	receptor activity	pathogenesis	integral to membrane
PFD0090c	-0.27	-6.28	1.71	-2.09	2.33	1.64	0.97	-3.49	7.09	0.016	Plasmodium exported protein (PHISTa), unknown function	1	null	null	null	null
PFD0440w	-0.05	-6.35	-0.69	1.89	-0.15	-4.82	0.00	-6.41	4.17	0.025	peptidase, M22 family, putative	1	yes	endopeptidase activity, zinc ion binding	null	null
PFD0460c	-0.72	2.49	-0.20	-4.94	0.13	-5.21	-0.16	-5.15	4.43	0.021	conserved Plasmodium protein, unknown function	0	null	null	null	null
PFD0625c	0.85	0.55	0.28	-5.09	0.12	-5.63	-0.30	-4.34	4.04	0.030	erythrocyte membrane protein 1, PfEMP1	0	null	receptor activity, cell adhesion molecule binding	cytoadherence to microvasculature, mediated by parasite protein, pathogenesis, rosetting, cell-cell adhesion, antigenic variation	integral to membrane, infected host cell surface knob, host cell plasma membrane
PFD0835c	-0.86	8.49	0.01	-5.98	-0.06	-5.53	-0.22	-1.94	4.31	0.001	LETM1-like protein, putative	1	null	null	null	apicoplast
PFD1005c	1.04	0.81	0.54	-3.82	0.17	-5.52	-0.50	-2.98	4.08	0.023	erythrocyte membrane protein 1, PfEMP1	0	null	receptor activity, cell adhesion molecule binding	pathogenesis, cytoadherence to microvasculature, mediated by parasite protein, rosetting, cell-cell adhesion, antigenic variation	integral to membrane, host cell plasma membrane, infected host cell surface knob
PFD1140w	-2.06	3.50	-0.38	-5.43	-0.16	-5.76	-0.12	-6.28	7.75	0.018	Plasmodium exported protein (PHISTc), unknown function	1	yes	null	null	null
PFE0050w	-1.14	0.22	-0.20	-5.72	-0.06	-5.88	-0.28	-5.43	6.73	0.044	Plasmodium exported protein, unknown function	1	null	null	null	null
PFE0055c	-0.18	-6.19	0.15	-5.85	-0.06	-5.88	-0.96	0.36	4.83	0.047	heat shock protein, putative	1	null	heat shock protein binding, unfolded protein binding	protein folding	membrane, apicoplast
PFE0135w	-0.85	-4.40	-0.33	-5.72	-0.08	-5.89	-1.54	0.36	7.69	0.036	DNAJ domain protein, putative	0	null	heat shock protein binding	null	null
PFE0285c	-1.11	0.14	-0.29	-5.45	-0.15	-5.67	0.26	-5.50	8.68	0.042	small ubiquitin-related modifier, putative	0	null	null	protein ubiquitination during ubiquitin-dependent protein catabolic process, modification-dependent protein catabolic process	null
PFE0420c	0.16	-5.82	0.05	-5.92	-0.15	-5.22	0.64	1.86	4.34	0.025	guanidine nucleotide exchange factor	0	null	Ran guanyl-nucleotide exchange factor activity	DNA packaging	nucleus, apicoplast
PFE0440w	0.04	-6.43	-0.05	-5.95	-0.28	-4.50	0.67	0.43	4.63	0.039	conserved Plasmodium protein, unknown function	0	null	null	null	null
PFE0595w	-0.59	0.73	-0.23	-4.72	-0.01	-5.91	0.03	-6.35	4.60	0.036	prefoldin subunit, putative	0	null	unfolded protein binding	protein folding	prefoldin complex
PFE0630c	-1.67	2.08	-0.49	-4.98	0.14	-5.78	-0.24	-5.87	5.44	0.024	orotate phosphoribosyltransferase	0	null	orotate phosphoribosyltransferase activity	nucleoside metabolic process	null
PFE0790c	-0.89	0.28	-0.28	-5.22	0.09	-5.79	0.16	-5.84	9.41	0.042	BolA-like protein, putative	0	null	null	null	null
PFE0885w	-0.62	0.40	-0.13	-5.61	0.14	-5.29	-0.33	-2.61	6.34	0.025	eukaryotictranslation initiation factor 3 subunit, putative	0	null	RNA binding, translation initiation factor activity	translational initiation	null
PFE1035c	-0.98	4.04	-0.09	-5.82	-0.03	-5.89	-0.28	-3.67	4.09	0.013	BIS(5'-nucleosyl)-tetraphosphatase (Diadenosine tetraphosphatase), putative	0	null	bis(5'-nucleosyl)-tetraphosphatase (asymmetrical) activity	null	null
PFE1125w	1.00	1.35	0.31	-4.99	0.16	-5.50	-0.36	-3.97	4.76	0.024	mitochondrial ribosomal protein L17 precursor, putative	0	yes	structural constituent of ribosome	translation	mitochondrial large ribosomal subunit
PFE1240w	-0.96	0.56	-0.02	-5.98	-0.07	-5.84	-0.23	-5.39	5.00	0.039	conserved protein, unknown function	0	null	iron-sulfur cluster binding, iron ion binding, catalytic activity	null	null
PFE1340w	-0.87	0.95	-0.12	-5.80	-0.05	-5.87	-0.39	-3.12	5.62	0.025	conserved Plasmodium protein, unknown function	2	yes	null	transport	membrane; integral to membrane
PFE1370w	-0.64	0.48	-0.52	-1.85	-0.08	-5.69	-0.18	-5.08	5.85	0.023	hsp70 interacting protein, putative	0	null	null	null	null
PFF0030c	0.58	-1.21	0.88	0.87	0.40	-2.72	-0.42	-2.01	4.17	0.011	erythrocyte membrane protein 1(PfEMP1) pseudogene	0	yes	null	null	null
PFF0080c	2.56	4.69	0.83	-4.03	-0.43	-5.02	0.51	-4.84	4.02	0.010	TPR-like domain containing protein, putative	0	null	null	null	null
PFF1365c	0.19	-5.93	0.10	-5.87	-0.14	-5.59	1.11	4.00	4.21	0.015	HECT-domain (ubiquitin-transferase), putative	2	null	acid-amino acid ligase activity	protein modification process	intracellular
PFI0355c	-0.16	-5.93	0.10	-5.83	0.24	-4.66	0.74	2.01	4.27	0.024	ATP-dependent heat shock protein, putative	0	null	protein binding, ATP binding, ATPase activity	null	HslUV protease complex
PFI0620w	-1.85	0.27	-0.09	-5.96	0.55	-4.82	-0.08	-6.38	5.25	0.043	conserved Plasmodium protein, unknown function	0	null	null	null	null
PFI0720w	-0.01	-6.46	-0.41	-4.63	-0.19	-5.41	-0.77	0.09	11.26	0.042	transporter (MFS family), putative	11	null	null	null	null
PFI0785c	0.21	-4.34	0.17	-4.86	-0.01	-5.91	0.60	4.68	4.17	0.011	sugar transporter, putative	11	null	transporter activity	transport	integral to membrane
PFI0920c	0.28	-5.89	0.25	-5.65	0.17	-5.67	-1.01	0.09	6.58	0.047	dihydrouridine synthase, putative	2	yes	FAD binding, tRNA dihydrouridine synthase activity	tRNA processing	apicoplast
PFI1155w	0.34	-4.98	-0.30	-5.13	0.00	-5.92	0.82	1.11	5.37	0.029	conserved Plasmodium protein, unknown function	0	null	cyclin-dependent protein kinase regulator activity	cell cycle	null
PFI1175c	-0.79	0.21	-0.31	-4.85	-0.38	-3.47	-0.26	-4.76	6.78	0.026	RNA binding protein, putative	0	null	nucleic acid binding	null	null

PFI1370c	-0.77	1.51	0.03	-5.97	-0.13	-5.41	0.38	-2.22	8.24	0.022	phosphatidylserine decarboxylase	0	null	phosphatidylserine decarboxylase activity	phospholipid biosynthetic process	null
PFI1570c	-1.99	5.94	-0.55	-4.11	0.16	-5.63	0.16	-6.05	7.46	0.006	M18 aspartyl aminopeptidase	0	null	aminopeptidase activity, zinc ion binding	proteolysis	vacuole
PFI1675w	-0.89	0.60	0.26	-5.26	-0.12	-5.67	0.12	-6.09	4.32	0.038	conserved Plasmodium protein, unknown function	0	null	null	null	null
PFI1775w	0.91	4.86	0.10	-5.72	0.17	-4.76	-0.21	-4.32	4.01	0.010	lysophospholipase, putative	0	null	null	null	null
PFL0015c	1.26	0.56	0.36	-5.28	0.31	-5.09	-0.50	-3.97	4.49	0.028	rifin	1	yes	molecular_function	antigenic variation	membrane, host cell plasma membrane
PFL1645w	-0.58	1.26	-0.24	-4.42	-0.07	-5.66	0.16	-4.97	5.29	0.025	conserved Plasmodium protein, unknown function	0	null	null	null	null
PFL1665c	-0.61	3.62	0.00	-5.98	-0.07	-5.56	-0.08	-5.78	4.60	0.017	conserved Plasmodium protein, unknown function	0	null	null	null	null
PFL1700c	0.23	-5.94	0.27	-5.46	-0.16	-5.60	0.98	1.36	5.13	0.030	V-type K+-independent H+-translocating inorganic pyrophosphatase, PfVP2	16	null	hydrogen-translocating pyrophosphatase activity, inorganic diphosphatase activity	proton transport	membrane
PFL1870c	0.28	-5.83	0.25	-5.61	0.02	-5.91	1.20	2.12	4.90	0.025	sphingomyelin phosphodiesterase, putative	0	null	null	null	null
PFL1960w	0.84	0.42	0.36	-4.55	0.14	-5.52	-0.29	-4.46	4.10	0.030	erythrocyte membrane protein 1, PfEMP1	0	null	cell adhesion molecule binding, host cell surface receptor binding, receptor activity	pathogenesis, cell-cell adhesion, antigenic variation, rosetting, cytoadherence to microvasculature, mediated by parasite protein	host cell plasma membrane, integral to membrane, infected host cell surface knob
PFL2275c	-1.45	1.10	-0.66	-4.15	-0.01	-5.92	0.45	-4.60	7.72	0.025	FK506-binding protein (FKBP)-type peptidyl-propyl isomerase	0	null	FK506 binding, peptidyl-prolyl cis-trans isomerase activity	protein folding	cellular_component
PFL2370c	-0.73	-2.27	-1.15	0.04	-0.21	-5.36	0.57	-2.49	6.87	0.021	conserved Plasmodium protein, unknown function	0	null	null	null	null
PFL2505c	0.19	-5.93	0.22	-5.45	-0.10	-5.74	0.85	1.90	4.60	0.025	rhoptry neck protein 3, putative	3	yes	molecular_function	biological_process	membrane, apicoplast
PFL2665c	1.06	1.65	0.49	-3.88	0.10	-5.77	-0.43	-3.34	4.34	0.021	erythrocyte membrane protein 1, PfEMP1	0	null	receptor activity, host cell surface receptor binding, cell adhesion molecule binding	antigenic variation, cytoadherence to microvasculature, mediated by parasite protein, rosetting, pathogenesis, cell-cell adhesion	integral to membrane, infected host cell surface knob, host cell plasma membrane
