# Supplementary material for: Genome Wide Adaptations of Plasmodium falciparum in Response to Lumefantrine Selective Drug Pressure
Source: PLoS One. 2012 Feb 27;7(2):e31623. doi: 10.1371/journal.pone.0031623 (PMC3288012; doi:10.1371/journal.pone.0031623)
Supplement: Table S2 — Known and putative transporters identified by microarray analyses to be differentially expressed during LM transient drug resistance acquisition. TM, transmembrane domains; SP signal peptide; AP apicoplast targeting signal; qP verified independently by qPCR; RNAseq optimal time of expression as determined by RNAseq [38]; linear modeling for each of the 4 time points as pair-wise comparisons (with log2 Ratio (L2R) and B value), EDGE p value, GO cellular component annotation; ref reference. (PDF) [file pone.0031623.s004.pdf]

Table S2. Known and putative transporters identified by microarray analyses to be differentially expressed during LM transient drug resistance acquisition. TM, transmembrane domains; SP signal peptide; AP apicoplast targeting signal; qP verified independently by qPCR; RNAseq optimal time of expression as determined by RNAseq [38]; linear modeling for each of the 4 time points as pair-wise comparisons (with log2 Ratio (L2R) and B value), EDGE *p* value, GO cellular component annotation; ref reference.

							Linear modeling-pairwise comparisons $V1S_{LM}$ vs.V1S											
							0h		12h		24h		36h		F-test	EDGE		
**ID**	**Product Description**	**TM**	**SP**	**api**	**qPCR**	**RNAseq [38]**	**log2Ratio**	**B**	**log2Ratio**	**B**	**log2Ratio**	**B**	**log2Ratio**	**B**	**adj.P.Val**	**P**	**GO Cellular Component**	**Ref.**
PFA0590w	ABC transporter, (CT family), putative, PfMRP1	11		x	x	24h	-0.08	-6.32	-0.33	-4.41	0.67	0.42	0.32	-3.60	0.027	0.021	parasite plasma membrane	[39]
PFC0725c	formate-nitrate transporter, putative	6				24h	-0.91	1.14	-0.17	-5.63	0.04	-5.88	-0.02	-6.41	0.035	0.543		[39]
PFC0840w	P-type ATPase, putative, PfATPase7	10				32-40h	0.33	-4.92	-0.26	-5.24	0.13	-5.63	0.69	0.12	0.038	0.016	parasite plasma membrane	[39]
PFE0825w	metabolite/drug transporter, putative	10			x	0 and 48h	-0.54	-2.27	-0.07	-5.91	0.11	-5.66	-0.28	-4.40	0.097	0.038	parasite plasma membrane	[39]
PFE1340w	conserved Plasmodium protein, unknown function	2	x			24-32h	-0.87	0.95	-0.12	-5.80	-0.05	-5.87	-0.39	-3.12	0.025	0.880	Golgi membrane	
PFE1455w	sugar transporter, putative	12				24 to 48h	-0.31	-2.63	-0.13	-5.37	-0.05	-5.78	-0.15	-4.88	0.119	0.008	parasite plasma membrane	[39]
PFE1525w	conserved Plasmodium membrane protein, unknown function	13	x		x	48h?	-0.16	-4.54	-0.03	-5.92	-0.04	-5.74	-0.02	-6.37	0.357	0.003	parasite plasma membrane	
MAL7P1.64	serpentine receptor, putative, PfSR25	8	x			0 and 24h	-0.70	3.26	-0.30	-3.48	-0.30	-2.38	-0.34	-1.26	0.008	0.669		[40]
PF08_0031	oxoglutarate/malate translocator protein, putative	1				24h	-1.49	0.01	-0.92	-3.50	0.11	-5.85	0.62	-4.04	0.028	0.000	mitochondrion inner membrane	
PFI0720w	transporter (MFS family), putative	11				0-8h	-0.01	-6.46	-0.41	-4.63	-0.19	-5.41	-0.77	0.09	0.042	0.023		[39]
PFI0785c	sugar transporter, putative	11		x		40h	0.21	-4.34	0.17	-4.86	-0.01	-5.91	0.60	4.68	0.011	0.024		[39]
PF10_0366	ADP/ATP transporter on adenylate translocase	3				24h	-0.99	0.98	-0.45	-4.21	-0.04	-5.89	-0.48	-2.74	0.023	0.111	mitochondrion inner membrane	
PF11_0172	folate/biopterin transporter, putative	11			x	40h	-0.63	-2.96	-0.16	-5.76	0.51	-3.19	0.84	0.44	0.023	0.013	parasite plasma membrane	[39]
PFL0220c	conserved Plasmodium membrane protein, unknown function	7				0 and 24h	-0.24	-0.79	-0.17	-3.38	-0.10	-4.39	0.01	-6.40	0.037	0.619	parasite plasma membrane	
PFL1700c	V-type K+-independent H+-translocating inorganic pyrophosphatase, PfVP2	16		x	x	40-48h	0.23	-5.94	0.27	-5.46	-0.16	-5.60	0.98	1.36	0.030	0.004	parasite plasma membrane	[39]
PF13_0300	mitochondrial inner membrane translocase, putative	2				24h	-0.94	0.27	-0.43	-4.43	0.23	-5.15	0.00	-6.41	0.036	0.099	mitochondrion inner membrane	
PF13_0358	mitochondrial import inner membrane translocase, putative	0				16h	-1.92	1.99	-0.40	-5.47	0.23	-5.64	-0.22	-6.08	0.025	0.161	mitochondrial intermembrane space	
MAL13P1.271	V-type ATPase, putative	4	x			16h	-0.85	1.49	-0.28	-4.90	0.08	-5.77	0.32	-3.70	0.023	0.172	vacuolar proton-transporting V-type ATPase complex	
