# Supplementary material for: Genome Wide Adaptations of Plasmodium falciparum in Response to Lumefantrine Selective Drug Pressure
Source: PLoS One. 2012 Feb 27;7(2):e31623. doi: 10.1371/journal.pone.0031623 (PMC3288012; doi:10.1371/journal.pone.0031623)
Supplement: Table S3 — qPCR analysis of PF10_0017 and PFB0105c using genomic DNA and cDNA templates from V1SLM and V1S P. falciparum isolates. cDNA samples were taken at the 36 h time point. All data were normalised to PF10_0210. (DOCX) [file pone.0031623.s005.docx]

**Table S3. qPCR analysis of PF10_0017 and PFB0105c using genomic DNA and cDNA templates from V1S_LM_ and V1S *P. falciparum* isolates.** cDNA samples were taken at the 36h time point. All data were normalised to PF10_0210.

| Gene | cDNA template | | | gDNA template | | |
| --- | --- | --- | --- | --- | --- | --- |
|  | Relative expression  V1S | Relative expression  V1S_LM_ | Ratio of expression  V1S_LM_/V1S | Estimated quantity  V1S  Mean (±SD) | Estimated quantity  V1S_LM_  Mean (±SD) | Ratio of expression  V1S_LM_/V1S |
| PF10_0017 | 0.02 (±0.01) | 1.47 (±0.59) | 73.5 | 0.11 (±0.08) | 0.65 (±0.33) | 5.90 |
| PFB0105c | 1.04 (±0.19) | 6.73 (±2.32) | 6.47 | 0.34 (±0.18) | 0.61 (±0.18) | 1.8 |
