# Supplementary material for: Genome Wide Adaptations of Plasmodium falciparum in Response to Lumefantrine Selective Drug Pressure
Source: PLoS One. 2012 Feb 27;7(2):e31623. doi: 10.1371/journal.pone.0031623 (PMC3288012; doi:10.1371/journal.pone.0031623)
Supplement: Table S4 — List of primers used for RT-qPCR and qPCR assays. (DOCX) [file pone.0031623.s006.docx]

**Table S4. List of primers used for RT-qPCR and qPCR assays.**

| **Gene ID** | **Primer sense** | **Sequence 5’ to 3’** |
| --- | --- | --- |
| PF10_0210 | forward | GCTGGAGGTGTGGGAGATT |
| PF10_0210 | reverse | GGAACAAGCGATGAAGAACC |
| PF11_0172 | forward | AATCGAAGCCATAACGGAAA |
| PF11_0172 | reverse | CTCAATATGGTGGCAAAAACA |
| PFL1700c | forward | TAGCTGACAATGCAGGAGGT |
| PFL1700c | reverse | AAGTGCAGCCGAACCTACAC |
| PFA0590w | forward | TGGCTTATAAATATTTGGGCATC |
| PFA0590w | reverse | TCCAAGGGTACTCAAAATTCG |
| PFE1525w | forward | CCAAGCGAATCAGACAAAAATAC |
| PFE1525w | reverse | TGCTCATTTTTCTCATCCACA |
| PFE0825w | forward | GCCAAATGACAAAACGAGTG |
| PFE0825w | reverse | AATCCCAATCCCAAGGCTAT |
| PF14_0331 | forward | GGCCAGAATTTTTAAGAAATGAA |
| PF14_0331 | reverse | TACCAGCAACAAATCCACCA |
| PFE1150w | forward | TGCCCACAGAATTGCATCTA |
| PFE1150w | reverse | TTCATCGTGTGTTCCATGTG |
| PFB0105c | forward | TGGTGGAAATGTTGTGGTCA |
| PFB0105c | reverse | TGTTGTCCATGAATGCTTTATCA |
| PF10_0017 | forward | AAGAAATTCCACCAAAACGTG |
| PF10_0017 | reverse | TCACAATCATATGGGTTATTC |
